# Hypothalamus Regulates Anabolic Metabolism of Articular Cartilage Superficial Chondrocytes through PGE2 Skeletal Interoception

**DOI:** 10.1002/advs.202501039

**Published:** 2025-03-26

**Authors:** Ziyi Wang, Xuequan Han, Jiawen Xu, Weixin Zhang, Kalp Patel, Jinjian Zheng, Mei Wan, Junying Zheng, Xu Cao

**Affiliations:** ^1^ Center for Musculoskeletal Research Department of Orthopedic Johns Hopkins University School of Medicine Baltimore MD 21205 USA; ^2^ Department of Biomedical Engineering Johns Hopkins University School of Medicine Baltimore MD 21205 USA

**Keywords:** articular cartilage, norepinephrine, PGE2, skeletal interoception, superficial zone

## Abstract

Degeneration of articular cartilage is the key underlying cause of most joint‐related diseases and yet little is known about its regeneration. Here, we report that skeletal interoception induces anabolic synthesis of superficial membrane by tuning down sympathetic norepinephrine (NE). Specifically, the superficial membrane is consumed during animal activity and anabolically renewed by the underneath chondrocytes in the superficial zone (SFZ). Notably, by stereotactic knockdown of sympathetic NE synthesis in the paraventricular nucleus, articular cartilage thickness increases. Moreover, deletion of the gene encoding the prostaglandin E2 (PGE2) receptor, EP4, in sensory nerves for ascending interoceptive pathway induces damage of superficial membrane and articular cartilage degeneration. In contrast, increase of interoceptive signaling by elevation of local PGE2 reduces sympathetic outflow to promote the anabolic renewal of superficial membrane. Importantly, inducible knockout of the β‐2‐adrenergic‐receptor (Adrb2) in the SFZ chondrocytes damages superficial membrane and treadmill running aggravates the damage. Mechanistically, NE‐mediated activation of Adrb2 induces internalization of Adrb2 and TGF‐β type II receptor as a complex, thereby regulating TGF‐β activity for articular cartilage homeostasis regeneration. Together, physical activity induces an anabolic renewal of the superficial membrane by downregulation hypothalamic NE for optimized thickness and integrity of articular cartilage.

## Graphical abstract

Hypothalamus sympathetic norepinephrine inhibits regeneration of articular cartilage. Mechanical loading on bone during animal activity elevates PGE2 skeletal interoception to promote an anabolic renewal of the superficial membrane by downregulation of norepinephrine for articular cartilage proper thickness and integrity.

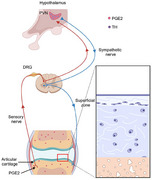

## 1 Introduction

Articular cartilage is a thin layer of connective tissue in diarthrodial joints that provides a lubricated surface to allow joints to move with low friction and to facilitate the transmission of mechanical loads to the underlying subchondral bone. Degeneration of articular cartilage leads to many skeletal disorders, such as osteoarthritis (OA) and spine degeneration, both of which can lead to severe pain and loss of mobility.^[^ ^1^ ^]^ As articular cartilage has no innervation or vascularization, its regeneration is often measured by thickness. The mechanism that maintains articular cartilage homeostasis is poorly understood. In particular, joint friction occurs at the surface of articular cartilage, and any loss of this surface needs to be continuously regenerated. However, little is known about this regenerative process. In brief, current therapies for articular cartilage degeneration include surgical replacement with artificial cartilage, pharmacological treatment to promote the expansion of articular cartilage thickness and pain management.^[^ ^2^ ^]^ With limited knowledge of articular cartilage regeneration, particularly, without knowing prostaglandin E2 (PGE2) skeletal interoception, the current treatment strategies pose limited effectiveness. Interestingly, previous studies reported that long‐term use of β blockers is associated with lower incidence of large joint arthritis, indicating a potential regulatory role of articular cartilage by the sympathetic nervous activity.^[^ ^3^ ^]^

The articular cartilage consists of three major layers: the superficial zone (SFZ), the deep zone, and the calcified zone.^[^ ^4^ ^]^ The SFZ has a superficial membrane that smoothens joint fiction, and it is marked by a high density of flattened cells underneath the membrane that have characteristics of progenitor cells.^[^ ^5^ ^]^ The SFZ maintains the proper lubrication of the joint by secreting lubricin, which is encoded by the gene *Prg4*.^[^ ^6^ ^]^ The SFZ bears the shear stress at the articular cartilage surface to maintain the homeostasis of articular joints. Compared with the SFZ, the deep zone cartilage contains thicker collagen fibrils and abundant proteoglycans to protect against compressive forces to facilitate the transmission of mechanical loads to the underlying subchondral bone of the joints and spine.^[^ ^4^ ^]^ The SFZ cells have been shown to regulate TGF‐β and lubricin to control joint homeostasis in response to mechanical loading.^[^ ^5^, ^6^, ^7^ ^]^ Therefore, the chondrocytes in the SFZ could potentially be critical for the regeneration of superficial membrane and deep zone articular cartilage.

Interoception is often described as the “sixth sense,” to transmit and sense the information of internal organ states whereas the classic five senses sight, smell, hearing, taste and touch all convey external information. Interoception is an emerging science and much remains to be explored.^[^ ^8^ ^]^ We recently found that PGE2 mediates skeletal interoception upon being secreted from osteoblasts in response to mechanical loading by inducing signaling of its receptor, EP4, in sensory nerves to activate cAMP‐response element binding protein (CREB) signaling in the hypothalamus.^[^ ^9^ ^]^ Central CREB‐signaling then downregulates sympathetic tone to induce osteoblastic differentiation, thus promoting bone formation.^[^ ^9^, ^10^ ^]^ In this manner a circuit of skeletal interoception is achieved that emanates from the periphery and acts via the hypothalamus to maintain proper bone homeostasis.^[^ ^11^ ^]^ Importantly, PGE2‐mediated skeletal interoception regulates weight bearing bone structure and metabolism in response to mechanical loading.^[^ ^12^ ^]^ Although, the role of interoception in skeletal homeostasis has now been well studied,^[^ ^11^ ^]^ its role in articular cartilage degeneration‐regeneration has not been studied at all as this structure is not innervated and thus a role for the central nervous system in its homeostasis has not been explored. Nonetheless, we investigated if skeletal interoception could regulate SFZ chondrocytes to promote regeneration of superficial membrane and articular cartilage homeostasis through integration of mechanical loads on the skeletal system. We found that it does, and thus these findings represent a paradigm shift in the understanding of articular cartilage and possibly in joint disease pathology.

## 2 Results

### 2.1 The Superficial Membrane Is Dynamically Consumed at Its Surface and Regenerated during Running

**Figure 1:**
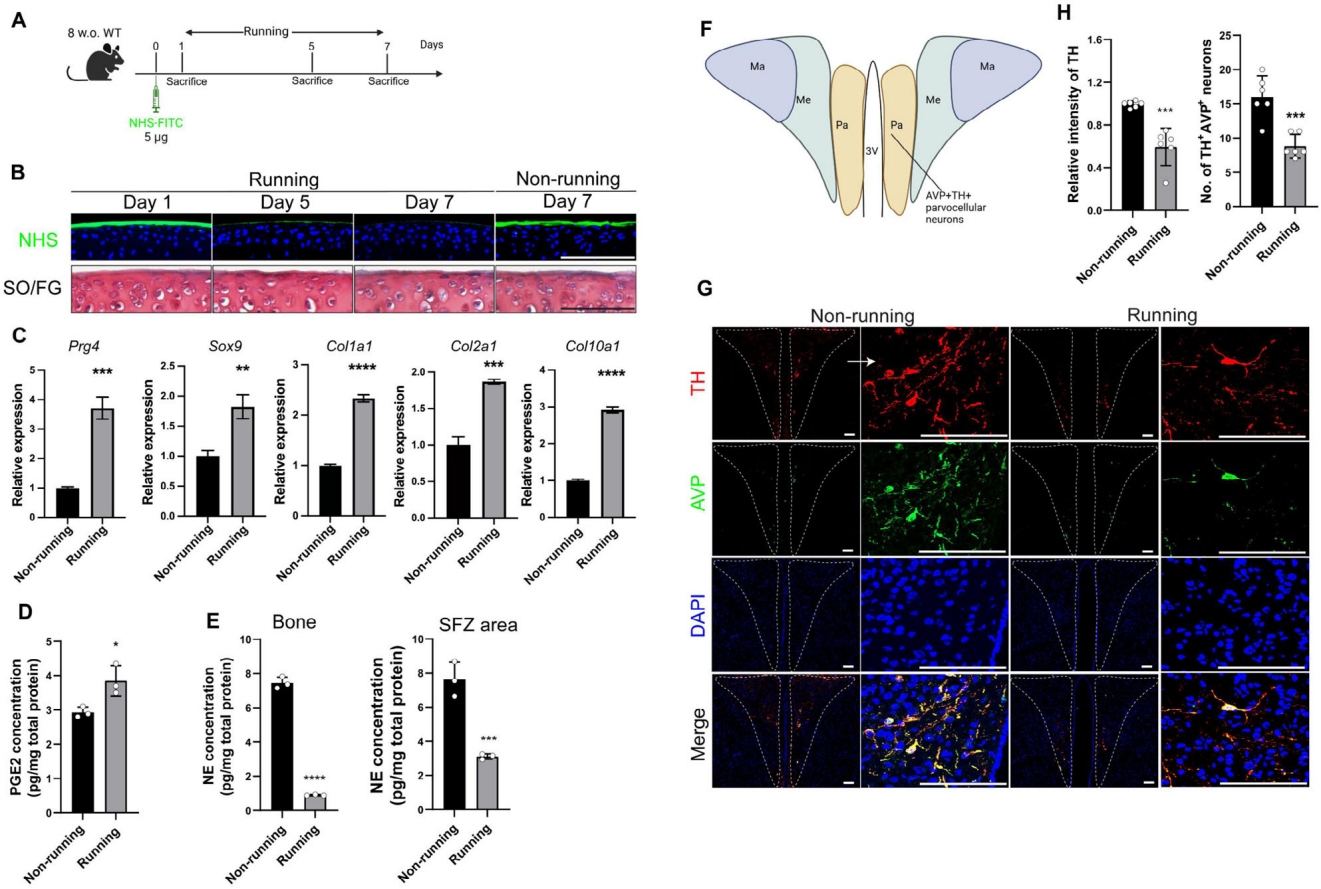
The superficial membrane of articular cartilage is consumed by friction force and constantly replaced by superficial zone chondrocytes. A) A schematic diagram illustrating the experimental design of using wild‐type (WT) mice at 8 weeks of age to label the superficial membrane of the articular cartilage by intra‐synovial injection of 5 µg of NHS‐FITC one day prior to being subjected to voluntary treadmill running for 1, 5, or 7 days. B) Representative images of fluorescent imaging of NHS labelling and safranin O/fast green staining of the knee joint of mice running for 1, 5, and 7 days. *n* = 6 each time point. Scale bars, 100 µm. C) RT‐qPCR analysis of the metabolic marker gene expression of superficial zone chondrocytes extracted from mice before and after 7 days of running. *n* = 3 each group. D) Measurement of PGE2 concentration in bone isolated from mice with or without running exercise for 7 days. *n* = 3 each group. E) Measurement of norepinephrine (NE) concentration in bone and SFZ area isolated from mice with or without running exercise for 7 days. *n* = 3 each group. F) Subdivisions of the PVN. Pa, parvocellular region; Ma, posterior magnocellular lateral region; Me, intermediocellular region. G,H) Representative images of tyrosine hydroxylase (TH) and vasopressin (AVP) staining in the periventricular nucleus (PVN) of F) the hypothalamus and G) their quantification. *n* = 6 each group. Scale bars, 100 µm.

To examine if the consumption of the superficial membrane at the joint surface from friction during running is dynamically regenerated by SFZ chondrocytes, this membrane was covalently labeled with *N*‐hydroxysuccinimide (NHS)‐FITC in two‐month‐old wild‐type (WT) mice (**Figure** **1**A). We then sacrificed the mice after they ran periodically on a treadmill for 1, 5, and 7 days and the knee joints were harvested. By florescent imaging of tissue sections from the knee joints we found that the superficial membrane of the tibia was well labeled on day 1 of running, but the labelled membrane was markedly consumed on day 5 and almost completely diminished on day 7 (Figure 1B). In contrast, the labeled superficial membrane was little consumed in the non‐running control group on day 7, indicating that running lead to the consumption of this membrane (Figure 1B). Importantly, by Safran O staining we demonstrated that the overall superficial membrane was maintained intact in the running group over the course of the 7 days (Figure 1B), suggesting that the consumption of the superficial membrane is dynamically regenerated by the underneath chondrocytes. Indeed, we found that the expression levels of the genes encoding lubricin, Sox9, type I, II and X collagen in the SFZ chondrocytes were significantly higher in the running group at day 7 relative to the non‐running group as determined by RT‐qPCR analysis of isolated superficial chondrocytes (Figure 1C). Notably, PGE2 in the tibia was significantly higher (Figure 1D), whereas norepinephrine (NE) levels were almost 9 times lower in tibia and significantly lower in the superficial zone of articular cartilage (Figure 1E). By immunostaining of PVN in hypothalamic sections (Figure 1F) we found that the levels of tyrosine hydroxylase (TH), a key enzyme in the biosynthetic pathway of NE, in PVN arginine‐vasopressin (AVP)‐positive neurons were significantly lower, which was associated with the higher levels of PGE2, in running mice relative to non‐running mice (Figure 1D,G,H), suggesting that skeletal interoception regulates NE levels at the superficial membrane, which diffuses into the bone from nearby nerve endings due to its porous nature.

### 2.2 Conditional Knockout of *Ptger* (EP4^−/−^) in Sensory Nerves Results in Loss of the SFZ

**Figure 2:**
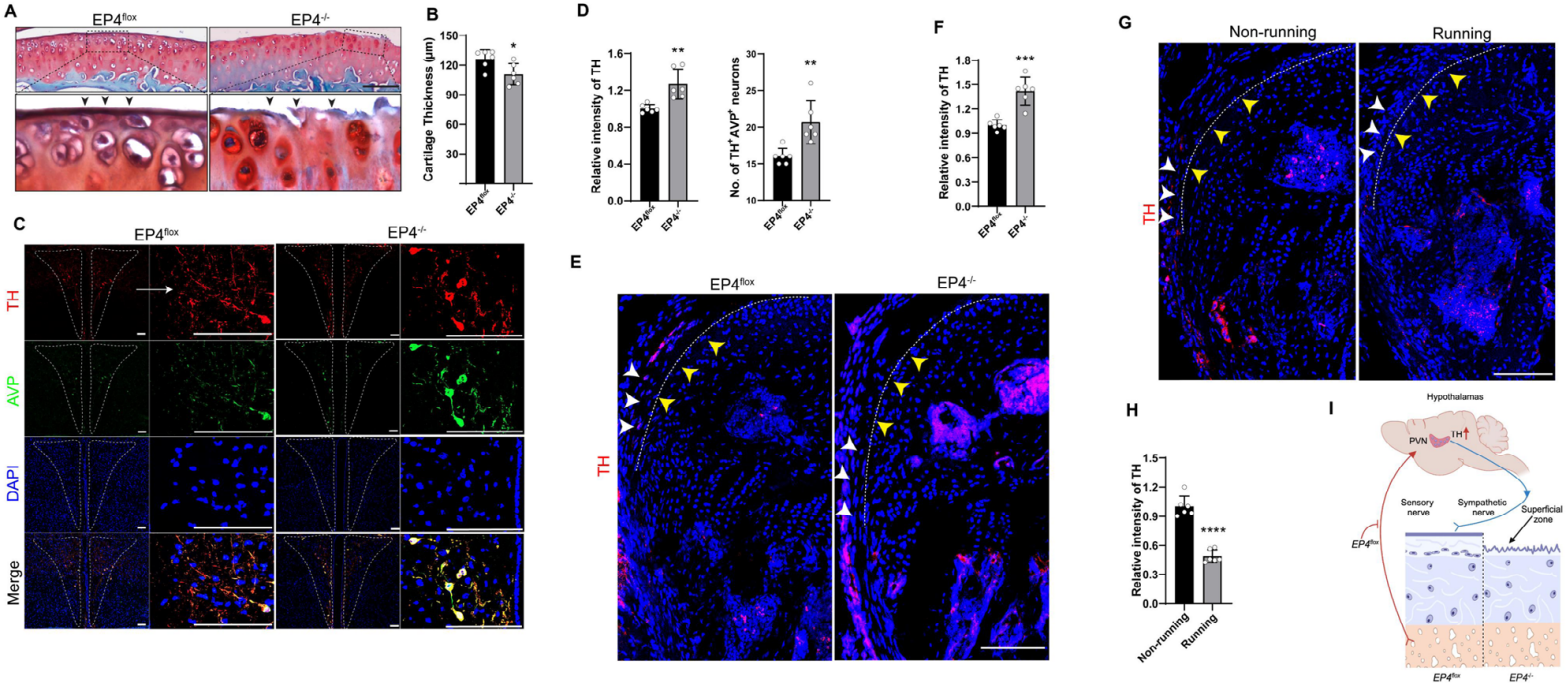
*Ptger4* deletion in sensory nerves induces degeneration of articular cartilage and loss of the SFZ. Representative Safranin O/fast green staining of the articular cartilage of A) EP4^flox^ and EP4^−^ *^/^* ^−^ mice and B) their quantitation. *n = *6 each group. Scale bar, 100 µm. Black arrows point toward superficial membrane of the articular cartilage. Representative images of TH staining in the PVN of C) the hypothalamus and D) quantitation. *n* *=* 6 per group. Scale bars, 100 µm. E,F) Representative images of immunostaining of TH in the junction area between cartilage and periosteum of E) the indicated mice strains and F) their quantitation. Yellow arrows point to superficial zone of articular cartilage and white arrows point to periosteum. *n* *=* 6 per group. Scale bar, 100 µm. Representative images of immunostaining of TH in the junction area between cartilage and periosteum of G) the non‐running and running mice and H) their quantitation. Yellow arrows point to superficial zone of articular cartilage and white arrows point to periosteum. *n* *=* 6 per group. Scale bar, 100 µm. I) A schematic diagram illustrating the findings that skeletal interoception regulates articular cartilage. Ascending interoception signaling initiated by PGE2 activates EP4 receptors on sensory nerves, which tones down sympathetic activity. The sympathetic neurotransmitter, NE, inhibits cartilage regeneration.

To examine if skeletal interoception regulates the regeneration of the superficial membrane, EP4^flox^ mice (*Ptger4^fl/fl^*) mice were crossed with *Avil‐*Cre mice to generate EP4^−/−^ (cKO) (*Ptger4^Avil^*) mice (Figure S1, Supporting Information). Thus, the ascending skeletal interoceptive signaling pathway was disrupted in EP4^−/−^ mice. By Safran O staining we found that the superficial membrane was entirely missing and the SFZ was largely lost in EP4^−/−^ mice compared to their EP4^flox^ littermates (**Figure** **2**A) and the thickness of the articular cartilage was significantly greater in the floxed control mice compared to the EP4^−/−^mice (Figure 2A,B). Most importantly, TH levels in the PVN AVP‐positive neurons were significantly upregulated in EP4^−/−^ mice compared with their floxed littermates (Figure 2C,D). As Calcitonin gene‐related peptide (CGRP)^+^ nerves represent a group of major innervation fibers in skeletal tissue and,^[^ ^13^, ^14^ ^]^ we then examined the change in CGRP^+^ sensory nerve fibers in the joint tissue. Deletion of *Ptger4* in the sensory nerves did not change the amount of CGRP^+^ sensory nerves in the joint tissue (Figure S2, Supporting Information).^[^ ^14^, ^15^ ^]^ Immunostaining of the junction tissue sections demonstrated that TH expression was remarkedly reduced in treadmill running mice relative non‐running mice, whereas TH level was significantly increased in EP4 mice (Figure 2E–H), suggesting that junction tissue could mediate interoceptive NE delivery from bone to articular cartilage. We further performed a surgical intervention to disrupt the junction of periosteum‐articular cartilage and found that NE dependent anabolic markers of the superficial zone chondrocytes were significantly reduced (Figure S3, Supporting Information). These results indicate that NE levels secreted in the bone are regulated by PGE2 ascending skeletal interoceptive activity in response to mechanical loading, which in turn controls the activity of SFZ chondrocytes for superficial membrane homeostasis (Figure 2I).

### 2.3 Stimulation of PGE2‐Mediated Skeletal Interoception Promotes Articular Cartilage Thickness

**Figure 3:**
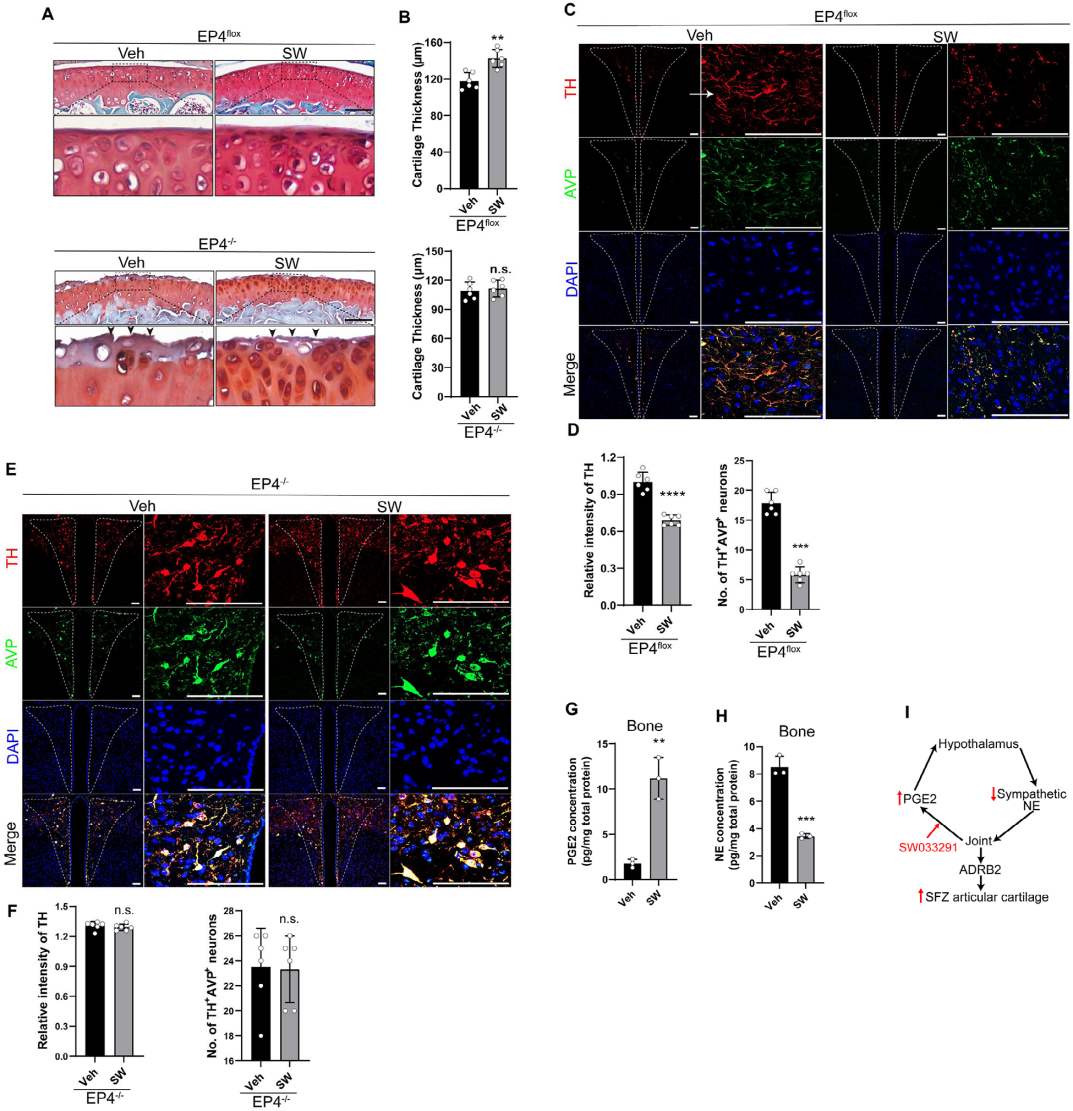
Increased PGE2 levels promote articular cartilage regeneration. Representative images of A) Safranin O/fast green staining of articular cartilage after EP4^flox^ or EP4^−/−^ mice received SW033291 (SW) treatment for 4 weeks and B) quantitation of the thickness of the articular cartilage. *n* *=* 6 each group. Scale bars, 100 µm. Black arrows point toward superficial membrane of the articular cartilage. Representative images of immunohistochemical staining of TH and AVP in the PVN of EP4^flox^ mice injected with C) SW or Veh and D) its quantitation. *n* *=* 6 per group. Scale bars, 100 µm. Representative images of immunohistochemical staining of TH and AVP in the PVN of EP4^−/−^ mice injected with E) SW or Veh and F) its quantitation. *n* *=* 6 per group. Scale bars, 100 µm. G) PGE2 level in the bone as measured by ELISA in mice treated with 10 mg kg^−1^ SW or Veh via i.p. injection every two days for 4 weeks. *n* *=* 3 per group. H) NE concentration in the bone of the indicated treatment groups. *n* *=* 3 per group. I) A schematic diagram illustrating the findings that increased PGE2 levels caused by SW injection promotes cartilage regeneration by reducing sympathetic activity.

To investigate if the articular cartilage regeneration is regulated by PGE2‐mediated skeletal interoception, we injected SW033291 (SW), a PGE2 degradation enzyme inhibitor, to increase local levels of PGE2 in the skeletal tissue. The thickness of the articular cartilage was significantly greater in the mice injected with SW every other day for 4 weeks relative to the vehicle‐treated mice (**Figure** **3**A,B). However, the greater articular cartilage thickness induced by SW treatment was abolished in EP4 KO mice and the SFZ was almost completely lost (Figure 3A,B). Like with running, TH levels in the PVN AVP‐positive neurons were significantly downregulated in SW‐treated mice compared to vehicle‐treated mice (Figure 3C,D), but the decrease was upregulated in EP4 KO mice (Figure 3E,F). The density of CGRP^+^ sensory nerves remained unchanged in the joint synovial tissue of SW‐treated mice (Figure S4, Supporting Information). Consistent with this observation, PGE2 levels in the bone were also significantly increased, while NE levels were lower, in SW‐treated mice relative to vehicle‐treated mice (Figure 3G,H). Together, these results indicate that PGE2‐mediated ascending interoception downregulates sympathetic activity to regulate articular cartilage anabolic activity (Figure 3I).

### 2.4 Knockdown of *Th* in the PVN Promotes Regeneration of the Superficial Membrane

**Figure 4:**
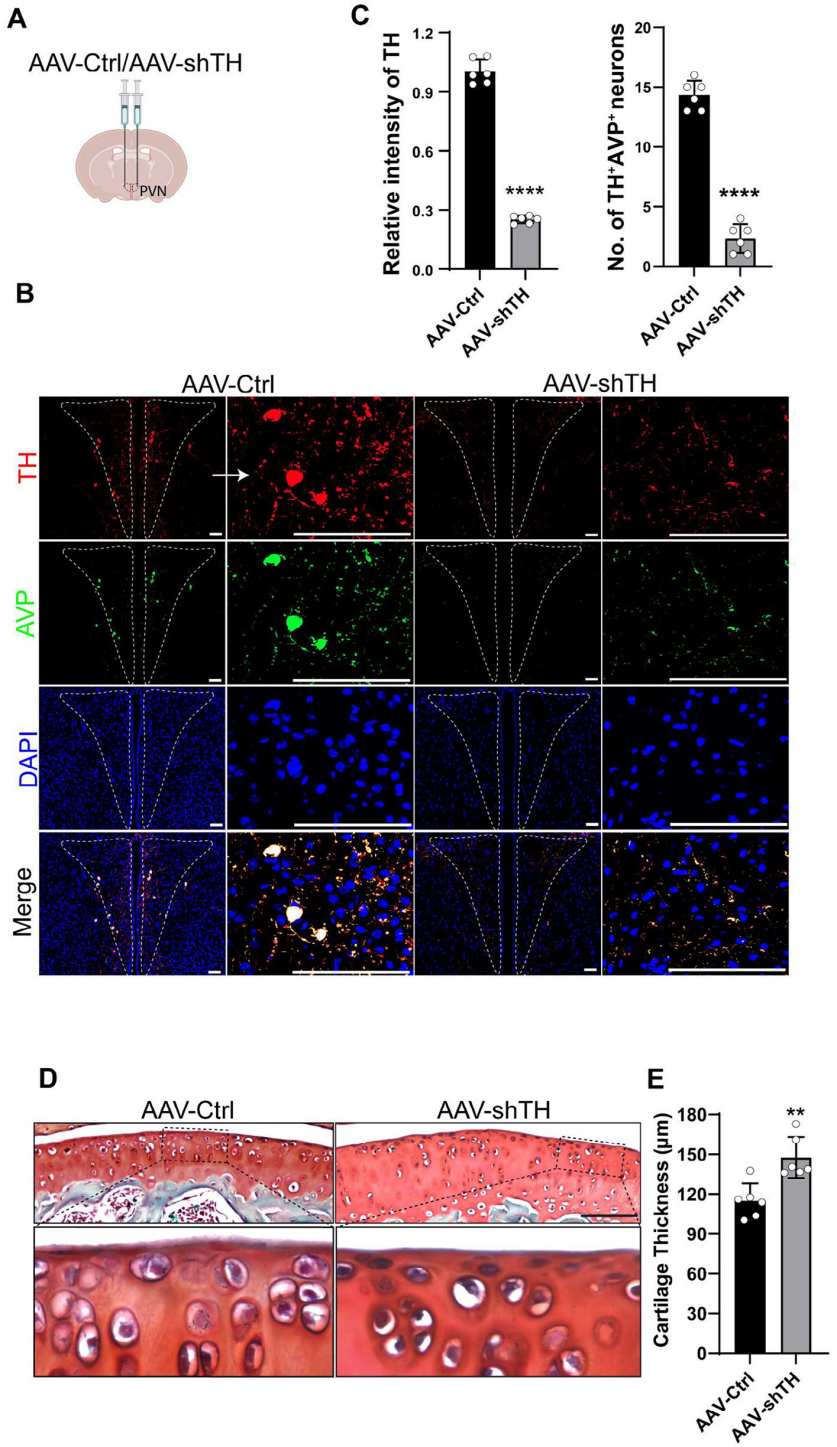
Stereotactic injection of AAV‐shTH into the PVN promotes regeneration of the articular cartilage. A) A schematic diagram illustrating the bilateral stereotactic injection of an AAV2 containing an shRNA against *Th* (AAV‐shTH) or a scrambled shRNA (AAV‐Ctrl) into the PVN. Representative images of immunostaining of TH and AVP in the PVN after Stereotactic injection of B) AAV‐Ctrl or AAV‐shTH and C) its quantitation. *n* *=* 6 per group. Scale bars, 100 µm. Representative images of Safranin O/fast green staining of D) the articular cartilage after stereotactic injection of the indicated AAV viruses and E) its quantitation. *n* *=* 6 each group. Scale bar, 100 µm.

To validate that the sympathetic tone originating from the PVN directly inhibits the regenerative activity of the SFZ of the articular cartilage, we performed bilateral stereotactic injection of an AAV2 virus expression an shRNA against *Th* (AAV‐shTh) or an AAV2 expressing a scrambled shRNA (AAV‐Ctrl) into the PVN and the mice were sacrificed 4 weeks after injection (**Figure** **4**A). By immunostaining we found that TH expression in the PVN was nearly abolished in AAV‐shTh group relative to AAV‐Ctrl group, indicating a success knockdown of *Th* expression in the hypothalamus (Figure 4B,C). Importantly, injection of AAV‐shTh in the PVN resulted in a significantly greater thickness of articular cartilage compared to the AAV‐Ctrl‐injected group (Figure 4D,E). Thus, these results validate the notion that skeletal interoception‐induced sympathetic tone negatively regulates the regeneration of articular cartilage, while blocking this signaling could increase the thickness of articular cartilage.

### 2.5 Conditional Knockout of *Adrb2* in the SFZ Chondrocytes Induces Loss of the SFZ

**Figure 5:**
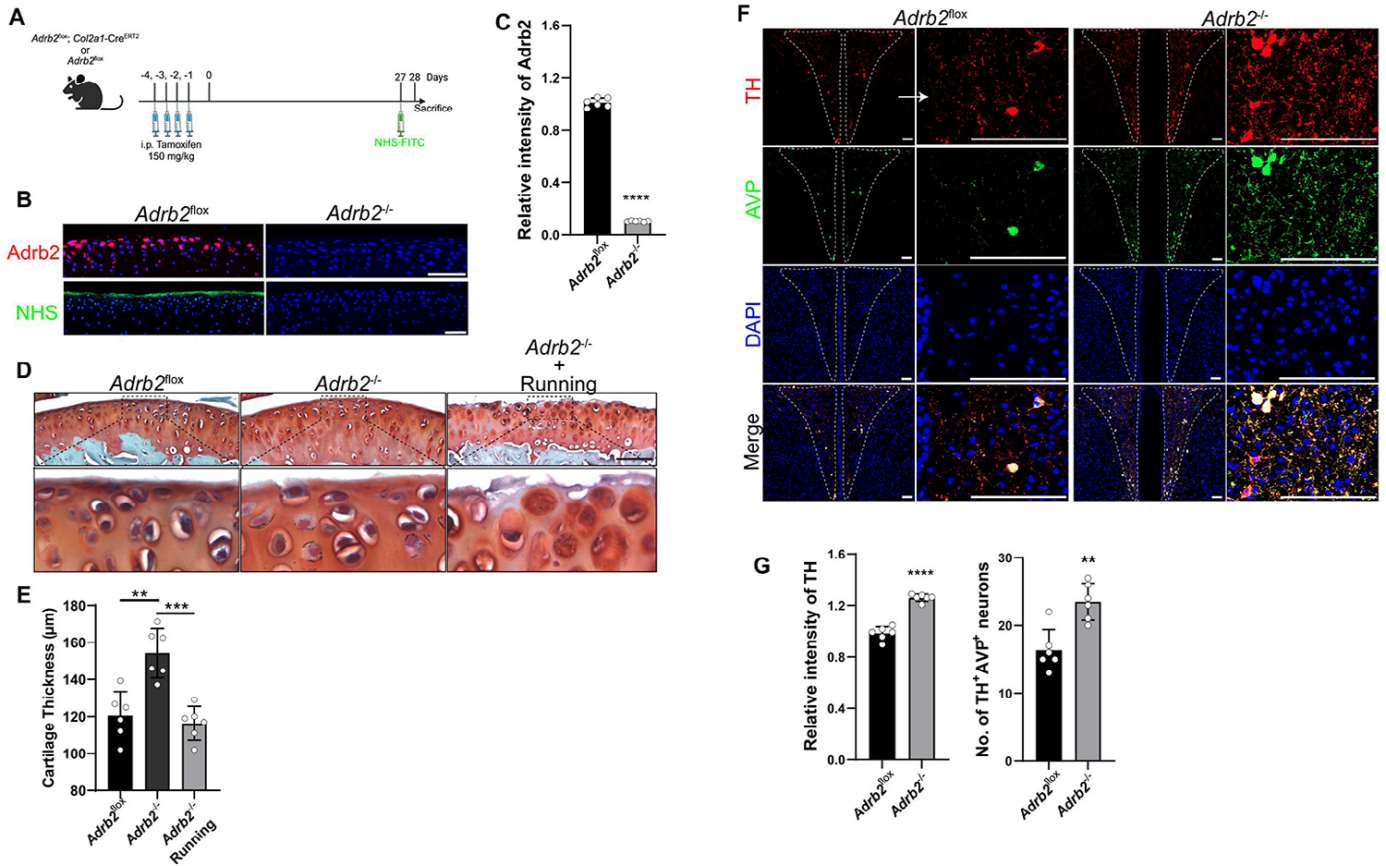
Conditional knockout of β2 adrenergic receptor (*Adrb2*) in SFZ chondrocytes disrupts the superficial membrane. A) A schematic diagram illustrating that *Col2a1*‐cre^ERT2^; *Adrb2* ^flox^ or *Adrb2* ^−/−^ mice were injected with tamoxifen at a concentration of 150 mg kg^−1^ for 4 consecutive days. Then, the mice were kept for 21 days before subjected to voluntary treadmill running for 7 days. B) Representative images of immunostaining of Adrb2 and fluorescent imaging of NHS in *Adrb2* ^flox^ and *Adrb2* ^−/−^ mice. Scale bars, 100 µm. C) Statistical analysis of relative staining of Adrb2 in articular cartilage. *n* = 6 each group. Representative images of D) Safranin O/fast green staining of the articular cartilage of the mice of indicated groups and E) quantification. *n =* 6 each group. Scale bar, 100 µm. Representative images of F) immunostaining of TH and AVP in the PVN of *Adrb2* ^flox^ and *Adrb2* ^−/−^ mice and G) quantification. *n* = 6 each group. Scale bars, 100 µm.

To validate that NE can directly regulate SFZ chondrocytes, we crossed NE receptor *Adrb2^f/f^* mice with *Col2a1‐CRE^ERT2^* mice to generate inducible *Adrb2* KO (*Adrb2^f/f^*: *Col2a1‐CRE^ERT2^*) mice in the chondrocytes (**Figures** **5**A and S5, Supporting Information). By immunostaining we found that Adrb2 is primarily expressed in the SFZ chondrocytes, and the expression was abolished in the *Adrb2* ^−/−^ (Figure 5B,C). Notably, the superficial membrane could not be covalently labeled by NHS‐FITC in *Adrb2* ^−/−^ mice (Figure 5B), indicating damage of the superficial membrane. Indeed, by Safran O staining we demonstrated that the superficial membrane was broken in *Adrb2* ^−/−^ mice with a slight increase in the thickness of articular cartilage. Interestingly, after running for a week, the SFZ was completely lost, and furthermore the thickness of articular cartilage dramatically decreased in *Adrb2* ^−/−^mice (Figure 5D,E). Analysis of a single‐cell RNA sequencing dataset (GSE172500) of WT mouse knee joint also showed that *Adrb2* is expressed mainly by the SFZ chondrocytes (Figure S6, Supporting Information).^[^ ^15^ ^]^ These results suggest that Adrb2 in the SFZ chondrocytes is essential for homeostasis of the superficial membrane and the integrity of the articular cartilage, while *Adrb2* deletion alters the structure of both the superficial membrane and the deep zone articular cartilage. The increased thickness of the cartilage could reflect a compensative effect through interoception. Indeed, TH levels in the PVN AVP‐positive neurons were significantly greater in *Adrb2* ^−/−^ mice compared with their floxed littermates (Figure 5F,G).

### 2.6 NE Regulates Anabolism of SFZ Chondrocytes by Inducing the Endocytosis of a TβRII‐Adrb2 Complex to Limit TGF‐β Signaling

**Figure 6:**
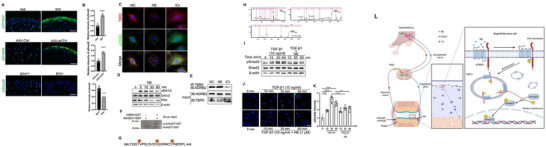
NE‐induced endocytosis of Adrb2 and TβRII downregulates TGFb signaling and lubricin expression in SFZ chondrocytes. Representative images of pSmad2 immunostaining in the joint of A) the indicated groups of mice and B) their quantitation. *n* *=* 6 per group. Scale bars, 100 µm. C) Representative images of immunostaining of Adrb2 and TβRII in chondrocytes from the SFZ‐derived chondrocytes treated with NE or ICI for 3 h. Scale bar, 10 µm. D) Western blot analysis of p‐Erk1/2 and PKA. E) Western blot analysis of immunoprecipitation (IP) with an antibody against TβRII and blotting for Adrb2. F) Adrb2CT‐GST incubated with cTβRII‐GST for 15 min at room temperature and resolved on a 4–20% Bis‐Acrylamide gel. G) Illustration of phosphorylation sites of Adrb2CT by cTβRII. H) Spectrum of the phosphorylated peptides analyzed by mass spectrometry. The phosphorylation sites are labelled. I) Western blot analysis of pSmad2 levels of SFZ chondrocytes treated with TGF‐β1 and NE. Representative images of J) pSmad2 immunostaining in SFZ chondrocytes treated with TGF‐β1and NE at different time point and K) its quantification. *n* = 6 per time point. Scale bar, 20 µm. L) A schematic diagram illustrating the regulation of articular cartilage by the brain.

TGF‐β activity is essential for both articular cartilage and subchondral bone homeostasis.^[^ ^16^ ^]^ We then examined if Adrb2 regulates TGF‐β signaling in SFZ chondrocytes. By immunostaining TGF‐β’s downstream mediator, phosphorylated Smad2 (pSmad2), we found that the levels of pSmad2 were induced specifically in the SFZ chondrocytes of both SW‐treated mice and PVN *Th* knockdown mice, but we found lower levels in the EP4 KO mice, relative to their controls (**Figure** **6**A,B). To examine the molecular mechanism by which NE signaling affects TGF‐β activity, we tested if NE induces endocytosis of Adrb2 to tone down TGF‐β signaling. We treated articular cartilage chondrocytes isolated from the SFZ with 1 µm NE or 10  µm ICI118551 (denoted ICI), an Adrb2 antagonist, for 3 h. The chondrocytes were co‐immunostained with antibodies against Adrb2 or the TGF‐β type II receptor, TβRII, the receptor for TGF‐β signaling. We found that NE induced internalization of both receptors in a cluster whereas ICI treatment did not induce the clustering formation (Figure 6C). To confirm the endocytosis, we performed Western blotting and found that NE induced phosphorylation of Erk1/2 and reduced PKA level (Figure 6D). By immunoprecipitation (IP) with an antibody against TβRII and blotting with an Adrb2 antibody we found that NE induced a greater interaction between the two receptors whereas ICI inhibited their interaction (Figure 6E). ICI was also injected intrasynovially into mice and found the thickness of articular cartilage increased relative to vehicle group. Treadmill running damaged SFZ with thinner articular cartilage in the ICI‐treated mice. Moreover, SFZ chondrocytes were treated with NE and ICI. While NE inhibited the expression of chondrogenic genes, ICI promoted expression of these genes (Figure S7, Supporting Information). To further examine whether TβRII phosphorylates Adrb2 directly, we purified the native constitutive active TβRII kinase, a cytoplasmic domain of TβRII (cTβRII), and a kinase‐dead cTβRII‐K277R,^[^ ^17^ ^]^ and then examined their potential phosphorylation of Adrb2 cytoplasmic domain (Adrb2CT). We found there was a shifted band of Adrb2CT after incubation with cTβRII for 15 min (Figure 6F). Mass spectrometry analysis identified three phosphorylated sites at Thr_398_, Ser_406_ and Ser_412_ Adrb2CT by cTβRII in three independent experiments (Figure 6G,H).

By Western blotting analysis we further demonstrated that TGF‐β1 induced Smad2 phosphorylation in chondrocytes from the SFZ, whereas NE inhibited the phosphorylation in a time‐dependent manner (Figure 6 I). We confirmed this observation by immunofluorescent staining after NE treatment and found a lower degree of TGF‐β1‐induced pSmad2 levels, indicating that NE‐induced the internalization of the two receptors as a complex, resulting in the inhibition of phosphorylation of Smad2 by the TGF‐β receptor complex (Figure 6J,K). Therefore, NE signaling induces endocytosis of Adrb2 and TβRII in a complex to downregulate TGF‐β signaling, thus reducing regenerative activity of articular cartilage.

## 3 Discussion

The nature of articular cartilage structure provides for low friction at the surface of the joints to reduce the degree of mechanical stress transmitted to the bone during movement. To the best of our knowledge, we demonstrate for the first time that the superficial membrane of articular cartilage is consumed at its surface by joint friction, which is dynamically regenerated from below by anabolic matrix synthesis by SFZ chondrocytes. Our findings suggest that the distinct structure of the three layers of articular cartilage processes different functions as an integrated functional organization. More importantly, the anabolic activity of SFZ chondrocytes is regulated by hypothalamic‐derived NE through skeletal interoception and its regulation of sympathetic tone. As we have previously reported, mechanical loads on the skeleton determine the levels of PGE2 secreted by osteoblasts to modulate hypothalamic‐driven sympathetic outflow to the skeleton.^[^ ^12^, ^18^ ^]^ In the current study, we find that PGE2 skeletal interoception also regulates chondrocytes in the superficial zone by sympathetic NE. Moreover, PGE2 has been shown to be regenerative in different tissues and organs including the immune system and pain.^[^ ^12^, ^19^ ^]^ Therefore, PGE2 interoception could represent a fundamental interoception for the brain monitoring and regulation of internal organs. The interoceptive activity stimulated by mechanical loading on the skeleton regulates the anabolic metabolism of SFZ chondrocytes to maintain homeostasis of the superficial membrane. Thus, the surface loss of the superficial membrane due to joint friction is dynamically regenerated by SFZ chondrocytes by downregulation of sympathetic outflow via skeletal interoception. In this way, the amount of superficial membrane loss resulting from surface friction could be accurately regenerated by SFZ chondrocytes through brain interpretation of PGE2‐mediated skeletal interoception activity.

Along these lines, we found that Adrb2, which is the receptor for the sympathetic transmitter norepinephrine, is only expressed in the SFZ chondrocytes in the articular cartilage. Interestingly, specific deletion of *Adrb2* in chondrocytes results in loss of the superficial membrane and SFZ chondrocytes. Moreover, specific deletion of *Ptger* (the gene encoding EP4, the receptor for PGE2) in sensory nerves to disrupt skeletal interoception also induces loss of the superficial membrane and degeneration of articular cartilage with a decrease in its thickness, as marked by thinning of the SFZ. Skeletal interoception activity downregulates hypothalamus‐generated sympathetic norepinephrine outflow to stimulate SFZ chondrocyte anabolic matrix protein synthesis in the membrane in response to mechanical loads on the skeleton. As periosteum directly connects articular cartilage through a junction with very abundant nerve fibers and blood vessels, our immunostaining of the junction sections demonstrated that TH expression was remarkedly reduced in treadmill running mice. Interestingly, surgical disruption of the junction significantly reduced anabolic activity of superficial zone chondrocytes. Therefore, norepinephrine secreted by sympathetic nerve endings likely diffuses into the SFZ during movement.

Chondrocytes in the SFZ also regulate deep zone cartilage. Lubricin is expressed by chondrocytes in the SFZ, which is essential for the structure and regenerative activity of the articular cartilage.^[^ ^6b^ ^]^ Interestingly, sympathetic release of norepinephrine activates Adbr2 signaling in the chondrocytes upstream of lubricin expression, as in the articular cartilage, as Adbr2 is only expressed in SFZ chondrocytes. Increased skeletal interoception activity downregulates sympathetic tone, which in turn promotes lubricin expression in the SFZ chondrocytes, and diffusion of lubricin promotes matrix protein synthesis in the deep zone cartilage. In addition, TGF‐β is abundantly deposited in the extracellular matrix of the articular cartilage and mechanical stress determines its activation to maintain articular cartilage homeostasis.^[^ ^16^, ^20^ ^]^ More importantly, TGF‐β has been shown to transcriptionally enhance lubricin expression in articular cartilage chondrocytes.^[^ ^20d^ ^]^ Our data here demonstrates that sympathetic‐driven norepinephrine release induces internalization of a Adbr2‐TβRII complex to tone down TGFβ signaling, resulting in the downregulation of lubricin expression in chondrocytes. Thus, while skeletal interoception activity tunes down sympathetic NE to regulate anabolism of superficial membrane, it also induces downregulation of both TGF‐β and lubricin in the SFZ to maintain structural integrity of deep zone and calcified cartilage zone.

Along these lines we found that deletion of *Ptger* in sensory nerves increases sympathetic tone, resulting in reduced TGF‐β signaling and lubricin expression in the SFZ, leading to thinning of the articular cartilage. In contrast, elevation of local PGE2 levels by injection of SW stimulates both TGF‐β signaling and lubricin expression in the SFZ and increases the thickness of articular cartilage by toning down hypothalamic sympathetic tone. Likewise, inhibition of Adbr2 activity in the chondrocytes by ICI injection as another means to interfere with NE signaling increased the thickness of articular cartilage. Similarly, stereotactic inhibition of central TH expression in hypothalamic neurons as yet a further method to reduce NE signaling also increased the thickness of articular cartilage. Together these data suggest that any means to raise local PGE2 levels in the bone, such as by walking or exercise with mechanical loading, could increase ascending interoceptive activity to tune down hypothalamic tone to better maintain the thickness of the articular cartilage and thus the health of the joint.

The superficial membrane homeostasis is accurately regenerated through PGE2‐mediated skeletal interoception activity. Physiological range of local PGE2 concentration seems critical for superficial membrane regeneration,^[^ ^21^ ^]^ and have both local and interoceptive roles.^[^ ^22^ ^]^ Moreover, high levels of PGE2 induces nociceptive hypersensitivity for pain.^[^ ^19c^ ^]^ PGE2 contributes to pain sensitization in joints by activating EP4 receptors on sensory neurons, which lowers the threshold for pain perception.^[^ ^11^, ^12^, ^23^ ^]^ This activation leads to the upregulation of pain‐related ion channels, such as TRPV1 and Nav1.8, enhancing pain transmission in joint.^[^ ^23^, ^24^ ^]^ In a rat model of restraint stress, high‐level PGE2 generated in damaged tissues increased EP4 and TRPV1 expression in DRG neurons, and EP4 antagonists relieved both inflammatory and neuropathic pain.^[^ ^25^ ^]^ We found that both EP4 and TRPV1 expressions were significantly decreased with low‐dose celecoxib, suggesting that physiological levels of PGE2 promote bone formation in porous endplates in spinal degeneration to modify the disease through PGE2/EP4 skeletal interoception.^[^ ^12c^ ^]^

Degeneration of articular cartilage is at the center of the pathogenesis of major joint diseases, including osteoarthritis, spine degeneration and rheumatoid arthritis, among others, but currently there is no effective therapy for such degeneration. However, as our findings are involved in fundamental cartilage biology, neuroscience and joint physiology, no disease model or skeletal aging were not able to be included in the current study. This unmet clinical need is largely due to a poor understanding of the underlying mechanisms of articular cartilage regeneration. Our findings here will help clarify these mechanisms and will hopefully allow for the rational design of effective therapies that will delay, maintain, or even reverse the loss of articular cartilage thickness in joint pathologies.

## 4 Experimental Methods

### Animal Experiments

All animal experiments were performed in accordance with the approved protocol, MO21M276 by the ACUC of Johns Hopkins University School of Medicine. C57BL/6 mice were purchased from Jackson Laboratory. *Advilin*‐cre (*Avil*‐cre) mice were acquired from Dr. Xinzhong Dong (Johns Hopkins University).^[^ ^26^ ^]^ The *Ptger4^fl/fl^* (EP4^flox^) mice were gifted by Dr. Brian L. Kelsall (National Institutes of Health).^[^ ^27^ ^]^ The *Avil*‐cre mice were crossed with EP4^flox^ mice and after backcrossing, *Avil*‐cre; *Ptger4^fl/fl^* (EP4^−^ *^/^* ^−^) mice were generated in which *Ptger4* was conditionally knocked out in the sensory nerves. Littermate EP4^flox^ mice were used as controls. Mixed male and female EP4^flox^ and EP4^−^ *^/^* ^−^ mice at 12 weeks of age (12 w.o.) were used for this study. *Col2a1*‐Cre^ERT2^ mice were kindly provided by Dr. Susan Mackem (Center for cancer Research, NIH).^[^ ^28^ ^]^ *Adrb2^fl/fl^* (*Adrb2* ^flox^) mice were acquired from Dr. Gerard Karsenty (Columbia University).^[^ ^29^ ^]^ The *Col2a1*‐Cre^ERT2^ mice were crossed with *Adrb2^fl/fl^* mice to generate *Col2a1*‐Cre^ERT2;^ *Adrb2^fl/fl^* mice (*Adrb2* ^−/−^), in which *Adrb2* was conditionally knocked out in articular cartilage. Tamoxifen diluted in corn oil was injected into 12‐week‐old *Adrb2* ^flox^ or *Adrb2* ^−/−^ mice at a concentration of 150 mg kg^−1^ for 4 consecutive days to induce knockout of *Adrb2* and after 4 weeks, the mice were harvested for analysis. All mice were housed on a 12‐h light/dark cycle with unlimited access to food and drinking water.

For each treatment group, equal numbers of C57BL/6J mice were randomly divided into vehicle and treatment groups. No preliminary statistical analysis was performed to determine the sample size. A summary of the mouse treatment method is provided in Table S1 (Supporting Information). SW033291 (SW) is a 15‐PGOH inhibitor and is used in this study to increase the level of prostaglandin 2 (PGE2) in vivo.^[^ ^19a^ ^]^ Male and female C57BL/6J mice (12‐week‐old) were injected with 100 µL vehicle control (10% ethanol, 90% corn oil) or 10 mg kg^−1^ per day SW every two days for 4 weeks. Selective β‐2‐adrenergic receptor (Adrb2) antagonist, ICI‐118551 (ICI), was used in this study to inhibit Adrb2 activation by the sympathetic neurotransmitter norepinephrine (NE).^[^ ^30^ ^]^ Male and female C57BL/6J mice (12‐week‐old) were injected with 10 µL vehicle control (sterile 1 × PBS) or 5 nmol ICI into the joint cavity daily for 4 weeks.

AAV‐2 was used in this study to deliver the shRNA into the periventricular nucleus (PVN) of the hypothalamus. AAV particles containing shRNA against *Th* or scrambled shRNA were purchased from Origene (Rockville, MD). For setting up the stereotactic injection, the mouse was anesthetized with xylazine and ketamine cocktail. After the mouse was fully anesthetized, it was fixed to the stereotactic injection device. Then, the head of the mouse was shaved and sterilized with 10% povidone iodine. Injections of AAV particles were made to the following coordinate relative to Bregma: A/P −0.90 mm, D/V −4.90 mm, M/L ± 0.50 mm. A total of 10^9^ CFUs of AAV containing either scramble shRNA or shRNA‐TH in 1 µL was injected to each location at a rate of 300 nL min^−1^. After surgery, the mouse was placed on a warm plate to allow recovery. After 4 weeks, the mice were sacrificed for analysis.

### Labeling Superficial Zone of Articular Cartilage with NHS

*N*‐hydroxysuccinimide (NHS) conjugated with FITC was purchased from Thermo Fisher (Waltham, MA). NHS covalently binds to lysine residues on the polypeptide chain in alkaline condition (pH 9.0) and has been used for labelling and tracking ECM in studies.^[^ ^31^ ^]^ Briefly, stock solution was prepared by dissolving NHS‐FITC in DMSO to a final concentration of 25 mg mL^−1^. On the day of experiment, working solution was prepared by further diluting the stock solution in 0.1 mM bicarbonate buffer (pH 9.0) to a final concentration of 0.5 mg mL^−1^. Male and female 12‐week‐old mice were injected with 10 µL labeling solution directly into the synovial cavity. The mice were then returned to their cage or subjected to treadmill running.

### Treadmill Running

To accelerate the consumption of the superficial membrane of articular cartilage, treadmill running was adopted. Male and female mice (8‐week‐old) were randomly divided into two groups, running and non‐running group. For the running group, the mice were placed onto a treadmill with 10% inclination. After an adaptation period of one week, the mice were voluntarily running on the treadmill at a speed of 15 m min^−1^ for 2 h a day. The mice in the non‐running group were kept in the same room as the running group and were allowed to move freely in their cages. After 1, 5, and 7 days of treadmill running. The mice were sacrificed, and their knee joints were harvested for subsequent analysis.

### Histological Analysis

The mice were sacrificed via cardiac perfusion with 1 × PBS followed by 4% PFA. Hind limbs were dissected and post‐fixed in 4% PFA for overnight at 4 °C then washed thoroughly with running water to remove excess fixative then decalcified with 0.5 m EDTA (pH 7.4) at 4 °C with shaking for 14 days. The decalcification solution was changed daily. After decalcification, the limbs were washed with 1 × PBS followed by paraffin embedding. Using a microtome, 5‐µm sections of the knee joint were made and stained with Safranin‐O and fast green. After mounting, the sections were imaged using a standard light microscope. The articular cartilage thickness was then analyzed using Image J software (NIH, Bethesda, MD).

### Immunohistochemistry

The brain samples were dissected after cardiac perfusion, and post fixed in 4% PFA for overnight at 4 °C with gentle agitation. The brain samples and decalcified knee samples were washed with 1 × PBS for 8 h at room temperature before being serial dehydrated with 20% and 30% sucrose for 24 h each. After dehydration, the tissue samples were embedded in optimal cutting temperature (O.C.T.) compound on dry ice. Sections were made using a cryostat.

For immunostaining, sections were washed with 1 × PBS to remove O.C.T. compound. Unspecific binding was blocked by 5% normal donkey serum (NDS) in PBS‐Triton‐X100 (1%) for 30 min at room temperature. Then, diluted primary antibodies were applied to each section and incubated at 4 °C in a humidified chamber overnight. The next day, the sections were washed 3 times with PBS‐0.3% Triton‐X100, incubated with appropriate secondary antibodies for 90 min at room temperature then mounted with mounting media (Vectashield H‐1500, Vector Laboratories, Newark, CA). Antibodies used in this study are summarized in Table S2 (Supporting Information). For staining of the superficial zone of the articular cartilage, an extra antigen retrieval process was performed before blocking, where the sections were incubated with 1 mg mL^−1^ hyaluronidase in 1 × PBS at 37 °C for 30 min followed by incubation with 2 mg mL^−1^ pepsin (pH 2.0) at 37 °C for 30 min.^[^ ^32^ ^]^

Stained sections were imaged using Zeiss LSM 800 confocal microscope (Carl Zeiss Microscopy, Oberkochen, Germany) using 10 × or 20 × objectives. The immunostaining images were then quantified using ImageJ and Imaris software (v10.0, Oxford Instruments, Abington, UK).

### Extraction and Culture of Superficial Zone Chondrocytes

For the extraction of superficial zone chondrocytes, 3‐week‐old male and female mice were used. The mice were sacrificed, and the femurs were dissected from the mice. Then the cartilage of the femoral head was carefully removed under a dissection microscope. The isolated femoral heads were washed with PBS for three times. A mixture of enzyme was made fresh before use by mixing 2 mg mL^−1^ of Pronase (Millipore Sigma, St. Louis, MO) with 1 mg mL^−1^ of Liberase (Millipore Sigma, St. Louis, MO) in DMEM/F12 (Thermo Fisher, Waltham, MA) with the addition of 0.5% BSA. The enzyme mix was pre‐heated to 37 °C before use and the femoral head was digested in the enzyme mix for 10 min at 37 °C for 10 min with gentle shaking. After 10 min, the supernatant was discarded and fresh enzyme mix was added to the femoral heads, which were further digested for 30 min at 37 °C with shaking. The resulting supernatant was collected and filtered through a 100 µm straining. The cells were pelleted by centrifugation at 400 × *g* for 5 min. The cells were counted and plated at a concentration of 5 × 10^3^ per cm^2^ for further experiments.

### RT‐qPCR

Primary mouse superficial zone chondrocytes were seeded into 6 well plates at a concentration of 10^6^ cells per well. The cells were then cultured until full confluence was reached. The cells were serum‐starved overnight and then treated with 1 µm NE and 10 µm ICI for 3 h. The cells were then lysed using TRIzol reagent (Thermo Fisher, Waltham, MA). Total RNA was extracted according to the manufacturer's instructions. For the synthesis of single stranded Complementary DNA (cDNA), 1 µg of total RNA of each sample was reverse transcribed using the PrimeScript RT Master Mix Kit (Takara, San Jose, CA). RT‐qPCR was then performed using SYBR GreenMaster Mix (Thermo Fisher, Waltham, MA) in a QuantStudio 3 system with the following setup: initial separation of 95 °C for 5 min, followed by 40 cycles of [95 °C for 30 s, 60 °C for 30 s], then final extension of 72 °C for 5 min. Target‐gene expression was normalized to the mRNA level of *β‐actin*, and relative gene expression was assessed using the 2^−ΔΔCT^ method.

### Western Blot

Chondrocytes extracted from mouse superficial zone were seeded in 6‐well plates at a concentration of 10^6^ cells per well, then the cells were serum‐starved for overnight before stimulation. The cells were then treated with 10 ng mL^−1^ rTGF‐β1 with or without 1 µm NE for the stated times. After each timepoint has reached, the cells were lysed with RIPA buffer. Then, the whole cell lysate was subjected to SDS‐PAGE to separate the proteins. The proteins were then transferred to a nitrocellulose membrane. Unspecific bindings were blocked by 5% BSA in TBST. Primary antibodies were diluted in 1% BSA in TBST and the membranes were incubated with primary antibodies at 4 °C with gentle shaking overnight. Then, the membrane was washed thoroughly and incubated with designated HRP‐conjugated secondary antibodies for 1.5 h at room temperature with agitation. Finally, the protein bands were visualized using Pierce ECL Western Blotting Substrate (Thermo Fisher, Waltham, MA). The band intensities were analyzed using ImageJ software.

### Immunoprecipitation (IP)

Primary mouse superficial zone chondrocytes were seeded into 6‐well plates at a concentration of 10^6^ cells per well. After the cells were attached, the cells were serum starved for overnight and then treated with 1 µm NE or 10 µm ICI for 3 h. The cells were then lysed and used for immunoprecipitation (IP) using Pierce Classic IP Kit (Thermo Fisher, Waltham, MA) as per manufacturer's instructions. In brief, the cells were lysed using 300 µL of ice‐cold Lysis/Wash Buffer. Cell debris was removed by centrifugation at 13 000 × *g* for 15 min. Then, the total protein from each sample was pre‐cleared with the Control Agarose Resin. Protein concentrations of the pre‐cleared lysate were then measured using BCA method. For the following IP reaction, 1 mg of each protein lysate was combined with 10 µg of the IP antibody and incubated at 4 °C overnight to form the immune complex. The next day, the immune complex was then added to Pierce Protein A/G Agarose in a spin column and incubated at 4 °C for 1 h with gentle shaking. The resin was then washed with IP Lysis/Wash buffer and the samples were eluted with sample buffer. The eluted protein samples were analyzed using western blot.

### ELISA

To measure the concentration of PGE2 and NE in tissue, the mice were euthanized and the whole knee joint was dissected. Under a dissection microscope, the meniscus and bone of each sample was separated. RIPA buffer was added to each sample, which was snap frozen using liquid nitrogen and grinded. To prevent degradation of NE, EDTA was added to each sample to a final concentration of 1 mm. The samples were further lysed using ultrasonication. Then, the protein concentration of each sample was determined using BCA method. The concentrations of PGE2 and NE were then determined using the PGE2 ELISA kit (Cayman, Michigan, MI) and Norepinephrine ELISA kit (Novus, Centennial, CO) according to the manufacturer's instructions. Finally, the PGE2 and NE concentrations were normalized to each mg of total tissue protein.

### Immunocytochemistry

Superficial zone chondrocytes were seeded onto sterile coverslips in 24‐well plates at a concentration of 2 × 10^4^ cells per well. The cells were then cultured overnight, and serum‐starved for overnight. The cells were then stimulated with 1 µm NE or 10 µm ICI for 3 h or 10 ng mL^−1^ TGF‐β1 with or without 1 µm NE for stated times. After stimulation, the cells were fixed with 4% PFA for 10 min at room temperature, then permeabilized with 0.5% saponin in PBS for 5 min. The cells were then blocked using 5% BSA in PBS for 30 min at room temperature. The cells were then incubated with diluted primary antibody overnight at 4 °C. After thorough wash, the cells were incubated with diluted secondary antibody at room temperature for 1.5 h. Then the cells were counterstained with DAPI and mounted onto a slide using ProLong Glass Antifade Mounting Media (Thermo Fisher, Waltham, MA) and placed into a dark box to allow the mounting media to settle before imaging. Finally, the cells were imaged using a Zeiss LSM880 confocal microscope with a 63 × oil objective. The fluorescent intensity was quantified using ImageJ software.

### Cloning, Expression, and Purification of c‐Terminal Adrb2 (Adrb2CT) and cTβRII

The GST‐tagged Adrb2CT was designed and cloned by Origene (Rockville, MD). The GST‐tagged cTβRII was cloned as previously described.^[^ ^17^ ^]^ The GST‐tagged Adrb2CT and cTβRII were expressed in *E. coli* (BL21) DE3 Rossetta 2 (Millipore Sigma, St. Louis, MO). Then, the recombinant proteins were pulled down using glutathione‐agarose beads. In vitro kinase assay was performed in a reaction buffer containing 50 mm Tris‐HCl, pH 7.5, 10 mm MgCl_2,_ and 100 mm of ATP. The products were resolved on a 4–12% Bis‐acrylamide gel. The phosphorylation sites were detected via mass spectrometry at Creative Proteomics (Shirley, NY).

### Single‐Cell RNA Sequencing Analysis

A previously published single‐cell RNA sequencing dataset was used in this study to investigate the expression of *Adrb2* in different populations of chondrocytes in mouse articular cartilage.^[^ ^15^ ^]^ This dataset was generated by disassociation of knee joint into single cell in post‐traumatic osteoarthritis mouse model. For the analysis, only the healthy (Day 0) data were used. The analysis was performed in R 4.2.3 using Seurat v4 package.^[^ ^33^ ^]^ After loading the dataset and QC, unsupervised clustering was performed and the dimension reduction using uniform manifold approximation and projection (UMAP) was performed. The chondrocyte clusters were further subclustered. The expression of *Adrb2* was shown using the FeaturePlot function.

### Statistical Analysis

All experiments were repeated at least 3 times independently, unless otherwise stated. Quantitative data were presented as mean ± standard deviation. Statistical analyses were performed using GraphPad Prism 9.0 (GraphPad Software, Boston, MA) in a blind fashion. Unpaired Student's *t*‐test with equal variance assumption between samples was used to determine the statistical differences between groups. For comparisons between multiple groups, ANOVA test was used with Tukey's test for multiple comparisons. A *p*‐value < 0.05 was considered statistically significant. *p < 0.05, **p < 0.01, ***p < 0.001, ****p < 0.0001.

## Conflict of Interest

The authors declare no conflict of interest.

## Code Availability

No new code was generated in this study.

## Supporting information



Supporting Information

Supporting Information

Supporting Information

## Data Availability

No new data were generated in this study. Previously published data, GSE172500, were analyzed in our study.
