# Supplementary material for: Hypothalamus Regulates Anabolic Metabolism of Articular Cartilage Superficial Chondrocytes through PGE2 Skeletal Interoception
Source: Adv Sci (Weinh). 2025 Mar 26;12(19):2501039. doi: 10.1002/advs.202501039 (PMC12097074; doi:10.1002/advs.202501039)
Supplement: Supplementary file 1 — Supporting Information [file ADVS-12-2501039-s001.docx]

Supporting Information

**Hypothalamus regulates anabolic metabolism of articular cartilage superficial chondrocytes through PGE2 skeletal interoception**

*Ziyi Wang†, Xuequan Han†, Jiawen Xu†, Weixin Zhang, Kalp Patel, Jinjian Zheng, Mei Wan, Junying Zheng, and Xu Cao^*^*

†Authors contributed equally.

Z. Wang, X. Han, J. Xu, W, Zhang, K, Patel, J. Zheng, M. Wan, J. Zheng, X. Cao

Center for Musculoskeletal Research, Department of Orthopedic, Johns Hopkins University School of Medicine, Baltimore, Maryland 21205, USA

E-mail: xcao11@jhmi.edu

J. Xu, M. Wan, X. Cao

Department of Biomedical Engineering, Johns Hopkins University School of Medicine, Baltimore, Maryland 21205, USA

Keywords: Skeletal interoception, Superficial zone, articular cartilage, norepinephrine, PGE2


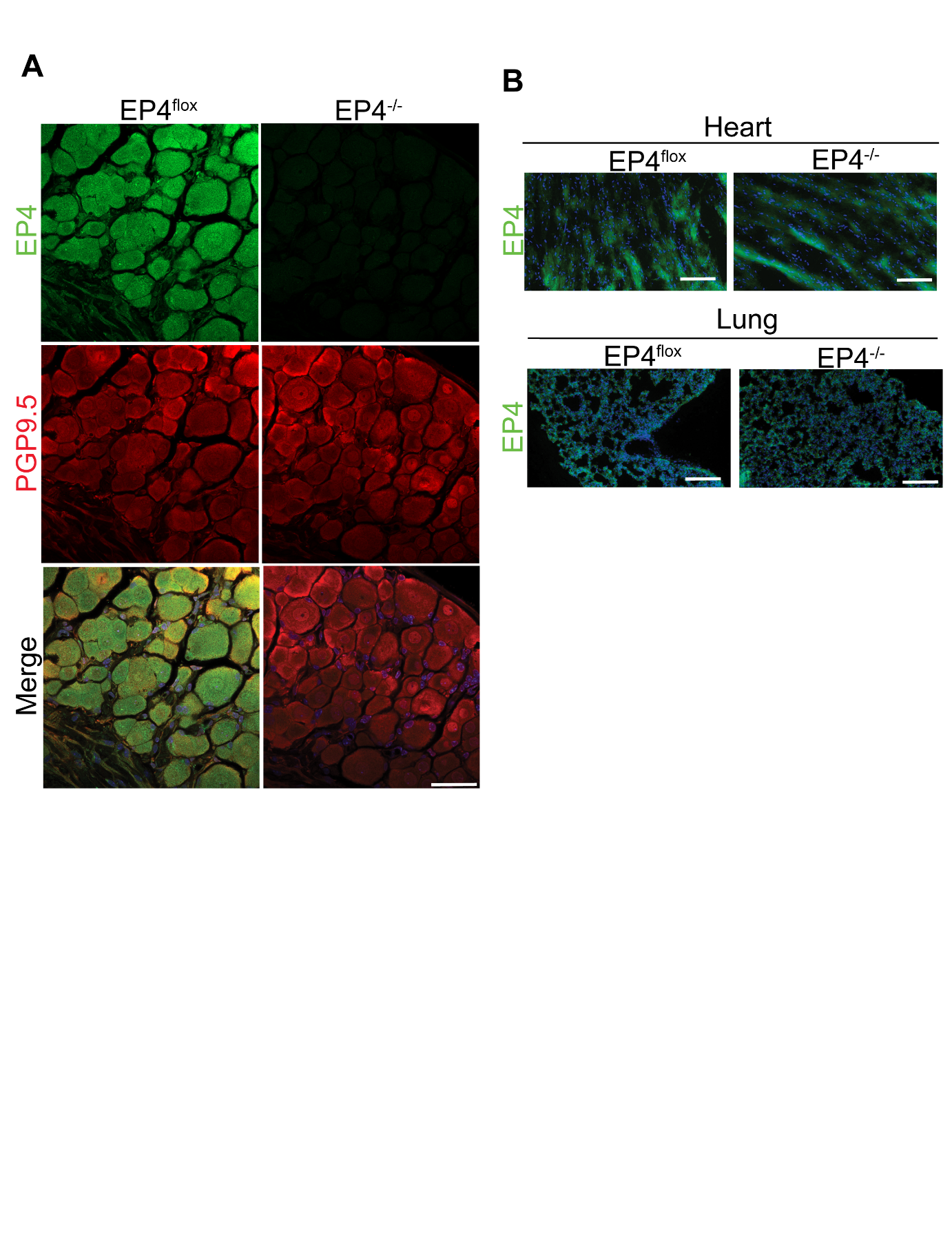


**Figure S1 Validation of EP4^-/-^ mice. (A)** Immunostaining of EP4 receptor and PGP9.5 in DRG isolated from EP4^flox^ and EP4^-/-^ mice. **(B)** Immunostaining of EP4 receptor in heart and lung isolated from EP4^flox^ and EP4^-/-^ mice. Scale bars, 20 µm.


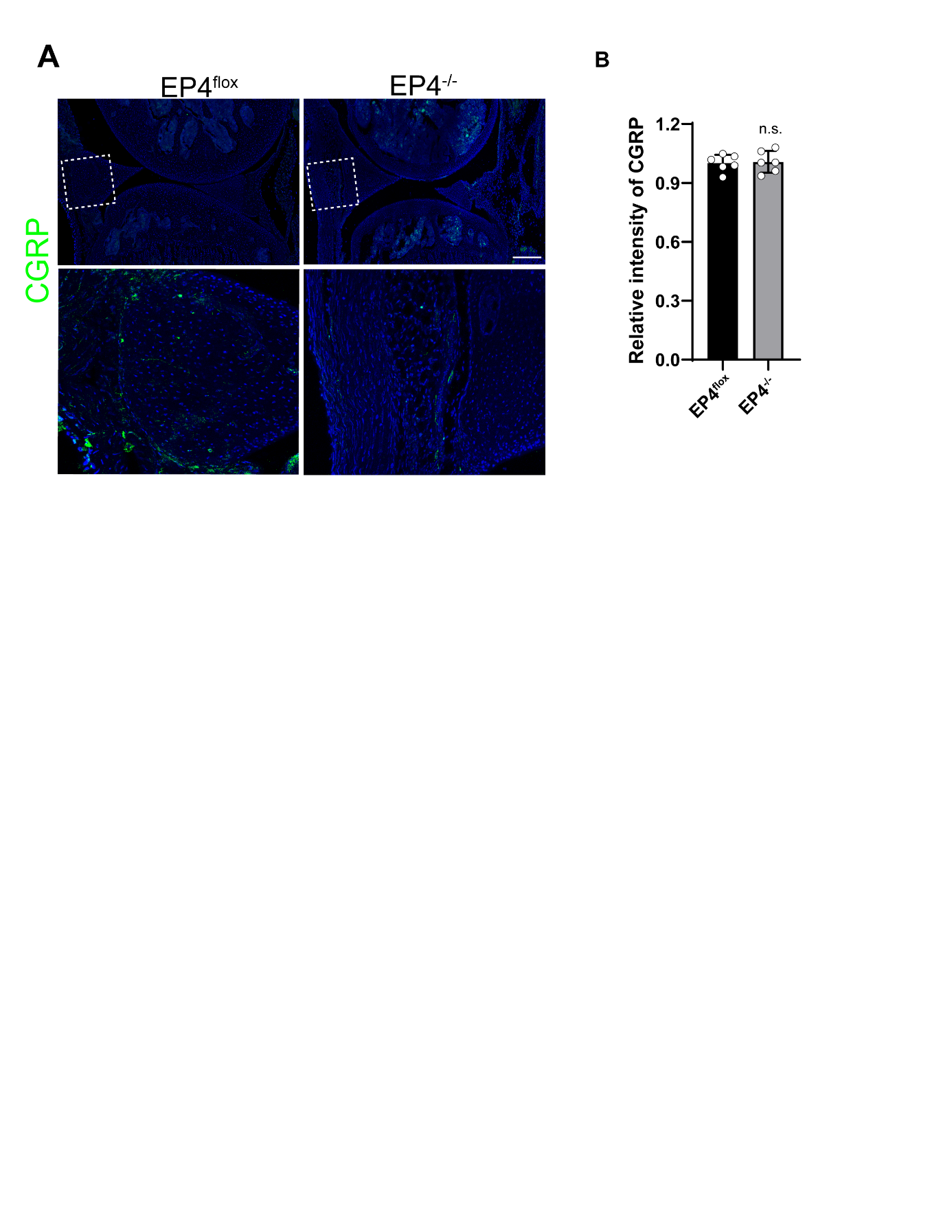


**Figure S2 Deletion of EP4 in the sensory nerve did not change sensory innervation in the mouse knee joints.** Immunostaining of CGRP with knee joint sections of EP4^flox^ and EP4^-/-^ mice. **(A)** Representative images of CGRP immunostaining in the knee joint of EP4^flox^ and EP4^-/-^ mice. **(B)** Statistical analysis of CGRP relative staining intensity. *n* = 6 per group. Scale bar, 20 µm.


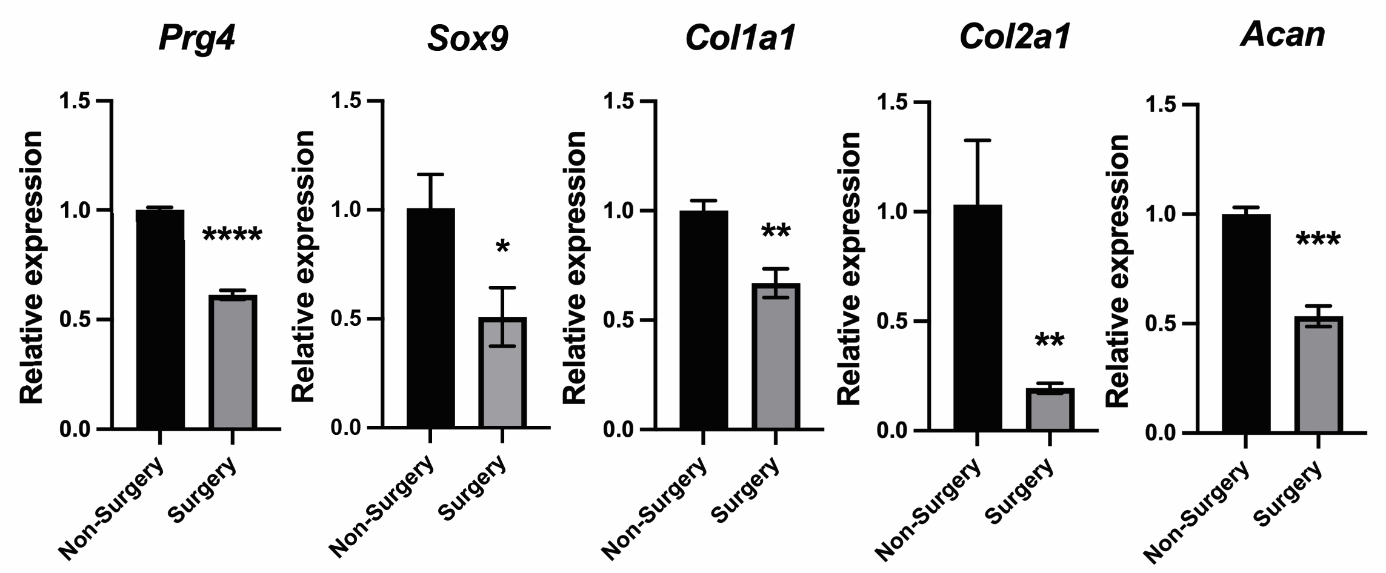


**Figure S3. Analysis of genetic expression of chondrocyte marker genes in SFZ cells after surgical disruption of the junction between periosteum and articular cartilage.** The SFZ cells were extracted 3 days after the junction between periosteum and articular cartilage were surgically disrupted. The expression of chondrogenic marker genes were examined by qPCR.


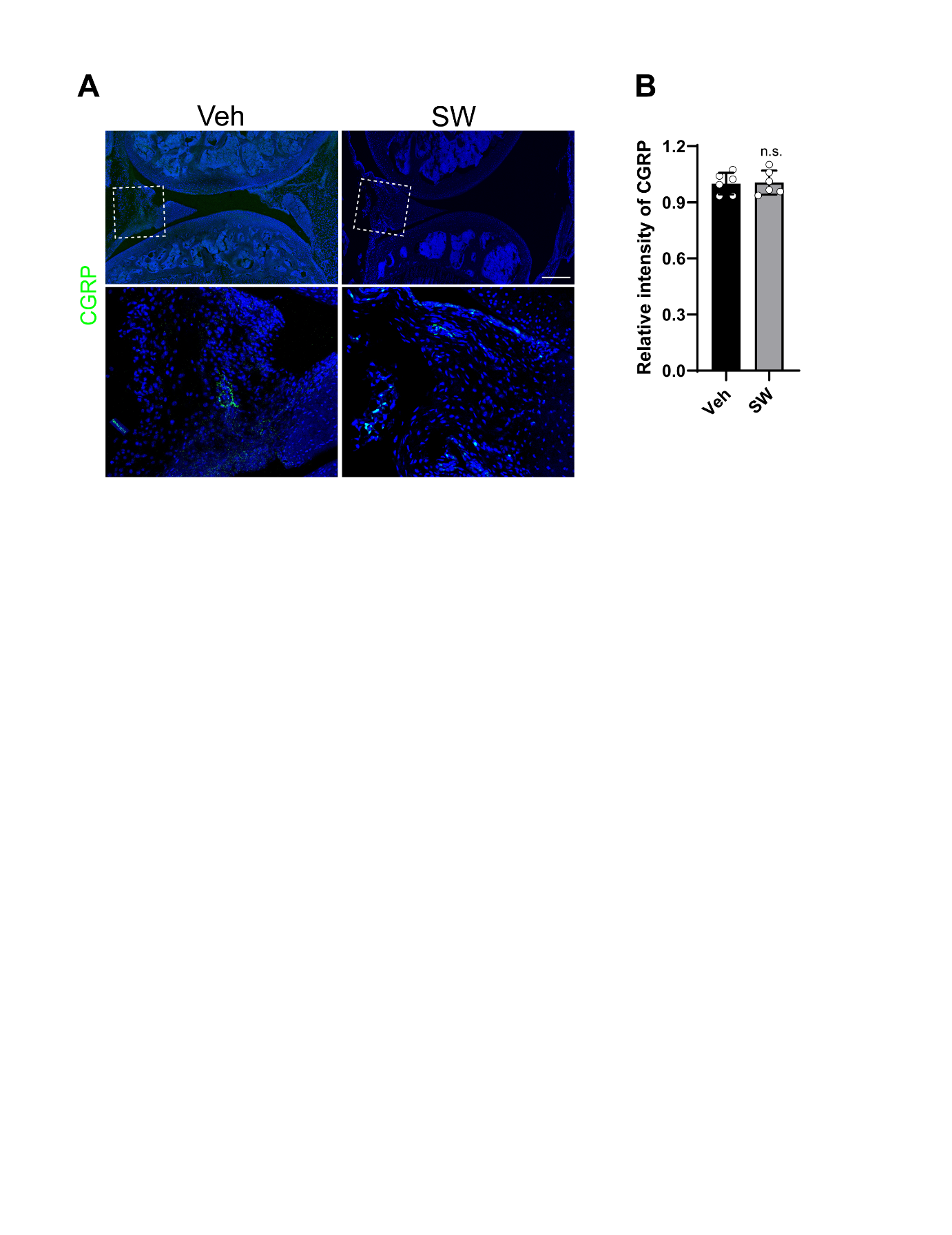


**Figure S4.** **Injection of SW033291 did not change the sensory nerve innervation in the knee joints.** Immunostaining of CGRP with knee joint sections of mice injected with SW033291 or vehicle. **(A)** Representative images of CGRP immunostaining in the knee joint in the Veh and SW033291 group. **(B)** Quantitative analysis of CGRP immunostaining intensity. *n* = 6 per group. Scale bar, 100 µm.


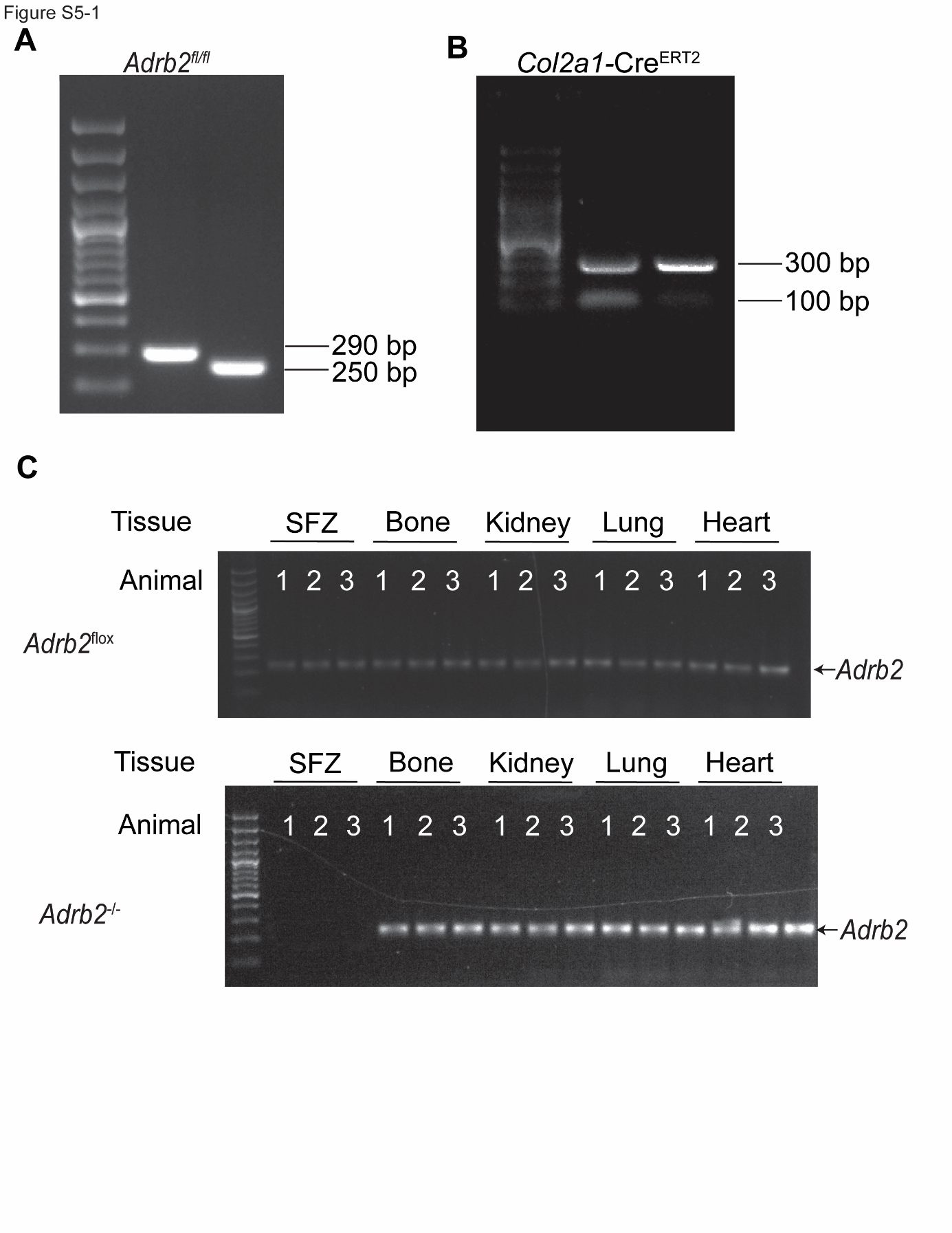


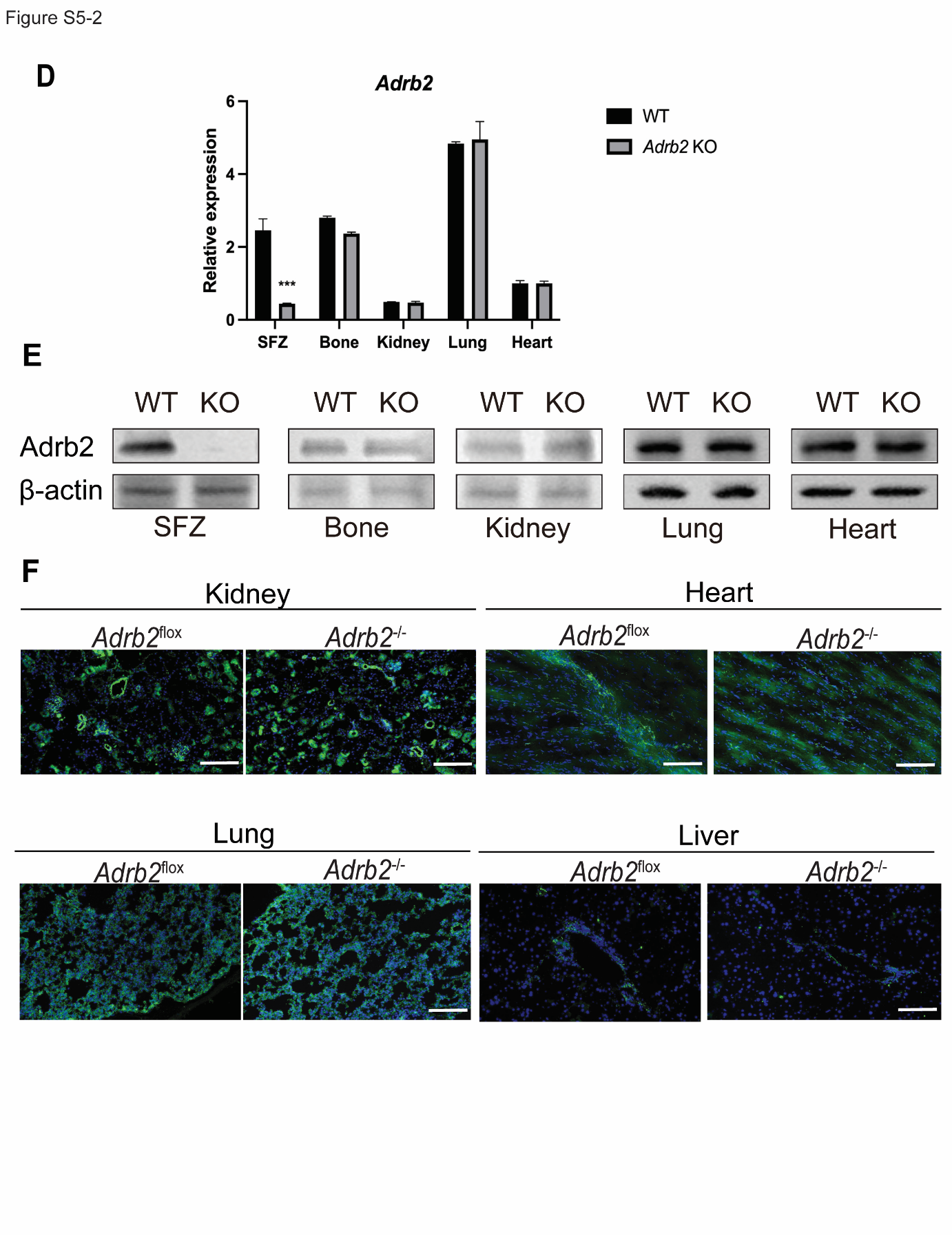


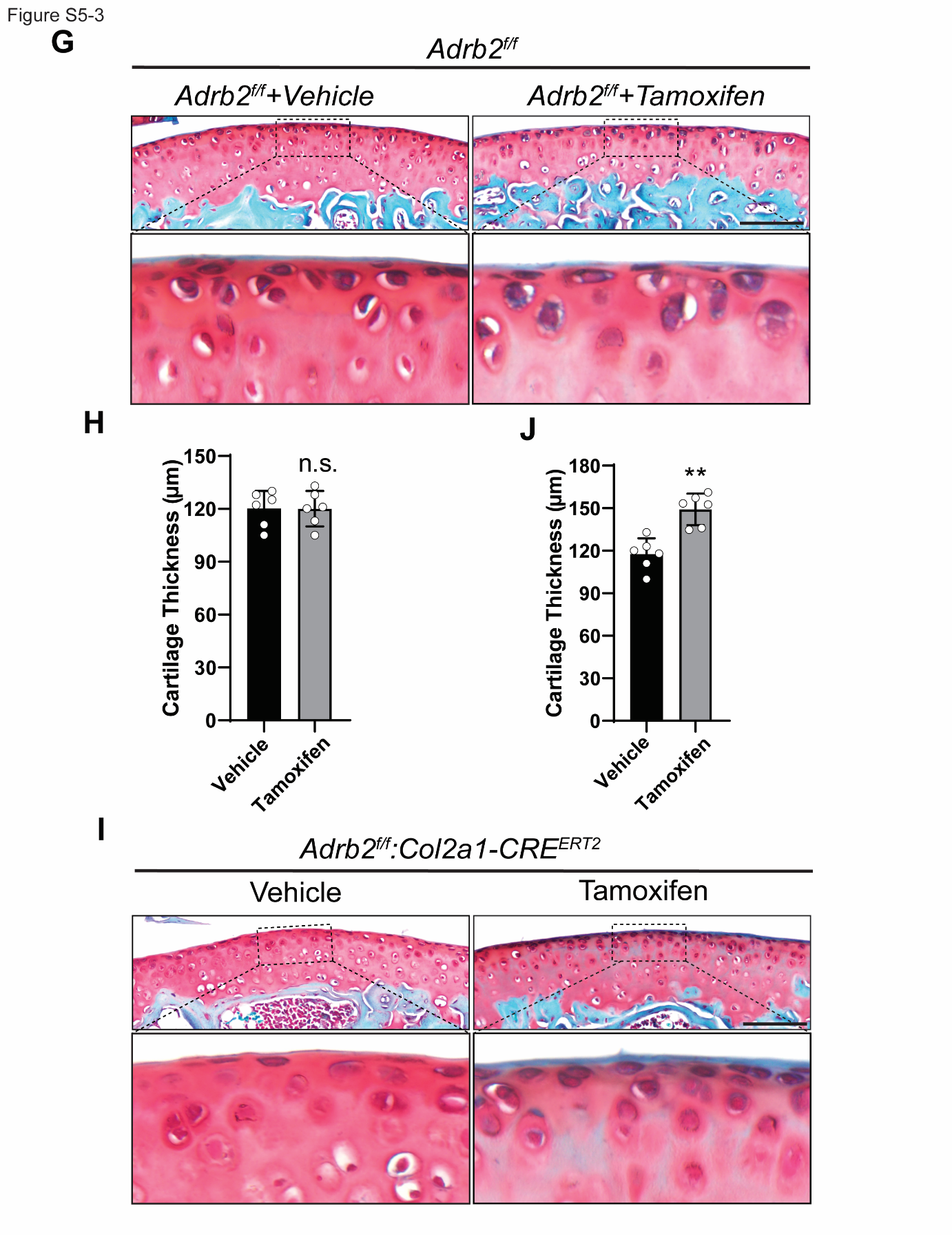


**Figure S5. Validation of inducible knockout of *Adrb2.* (A)** Genotyping of *Adrb2*^flox^ allele. Expected outcome: WT: 250 bp; Floxed: 290 bp. The PCR reaction was conducted with the following primers: *Adrb2*^flox^: Forward: 5'-AGCTGAGTGTGCAGGACGCA-3', Reverse: 5'-CGCTTCGTCCCGTTCCTGAGT-3'. **(B)** Genotyping of *Col2a1-*Cre allele. Expected outcome: WT: 300 bp; Cre: 100 and 300 bp. The PCR reaction was conducted with the following primers: Forward: 5'-GCGGTCTGGCAGTAAAAACTATC-3', Reverse: 5'-GTCAAACAGCATTGCTGTCACTT-3'. (**C, D**) RT-PCR analysis of *Adrb2* RNA levels in different tissues. Agarose gel electrophoresis of RT-PCR product (C) and the quantification (D). (**E**) Western blot analysis of Adrb2 and β-actin protein expression of different tissues from *Adrb2*^flox^ and *Adrb2*^-/-^ mice. (**F**) Representative images of immunostaining of Adrb2 in different tissues from *Adrb2*^flox^ and *Adrb2*^-/-^ mice. (**G, H**) Adrb2^flox^ mice were injected with Veh or 150 mg/ml of tamoxifen for 4 consecutive days and their articular cartilages were analyzed using Safranin O/fast green staining (G) and its quantification. n=6 per group. Scale bar, 100 μm. (**I, J**) *Adrb2*^flox^; Col2a1-cre^ERT2^ mice were injected with Veh or 150 mg/ml of tamoxifen for 4 consecutive days and their articular cartilages were analyzed using Safranin O/fast green staining 4 weeks after injection (I) and its quantification. n=6 per group. Scale bars, 100 μm.


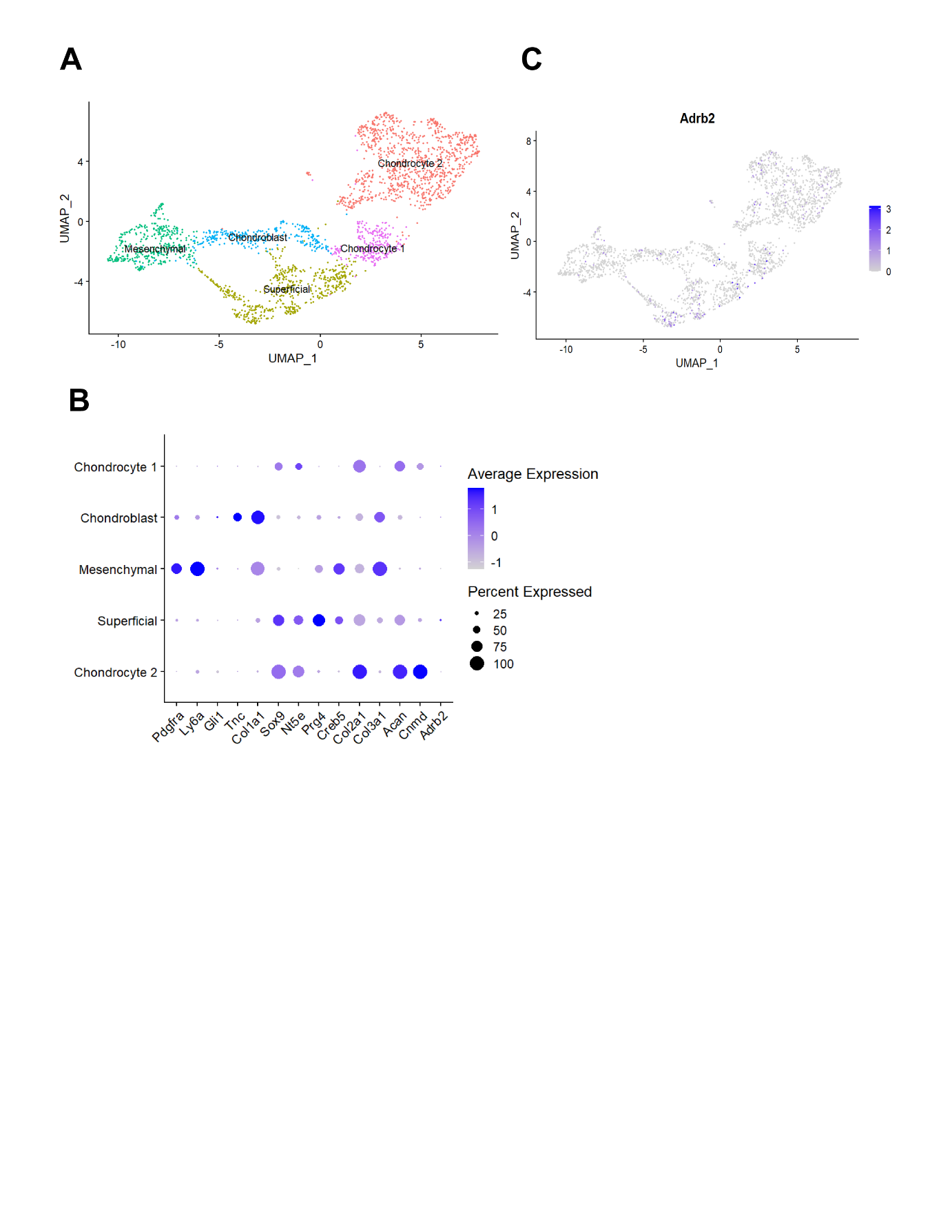


**Figure S6. ScRNA-seq analysis shows that superficial zone chondrocytes express *Adrb2.* (A)** Unsupervised clustering of chondrocytes resulted in 5 clusters, Mesenchymal, Chondroblast, Superficial, Chondrocyte 1 and Chondrocyte 2. **(B)** Expression profile of marker genes used to define the clusters. **(C)** Expression of *Adrb2*.


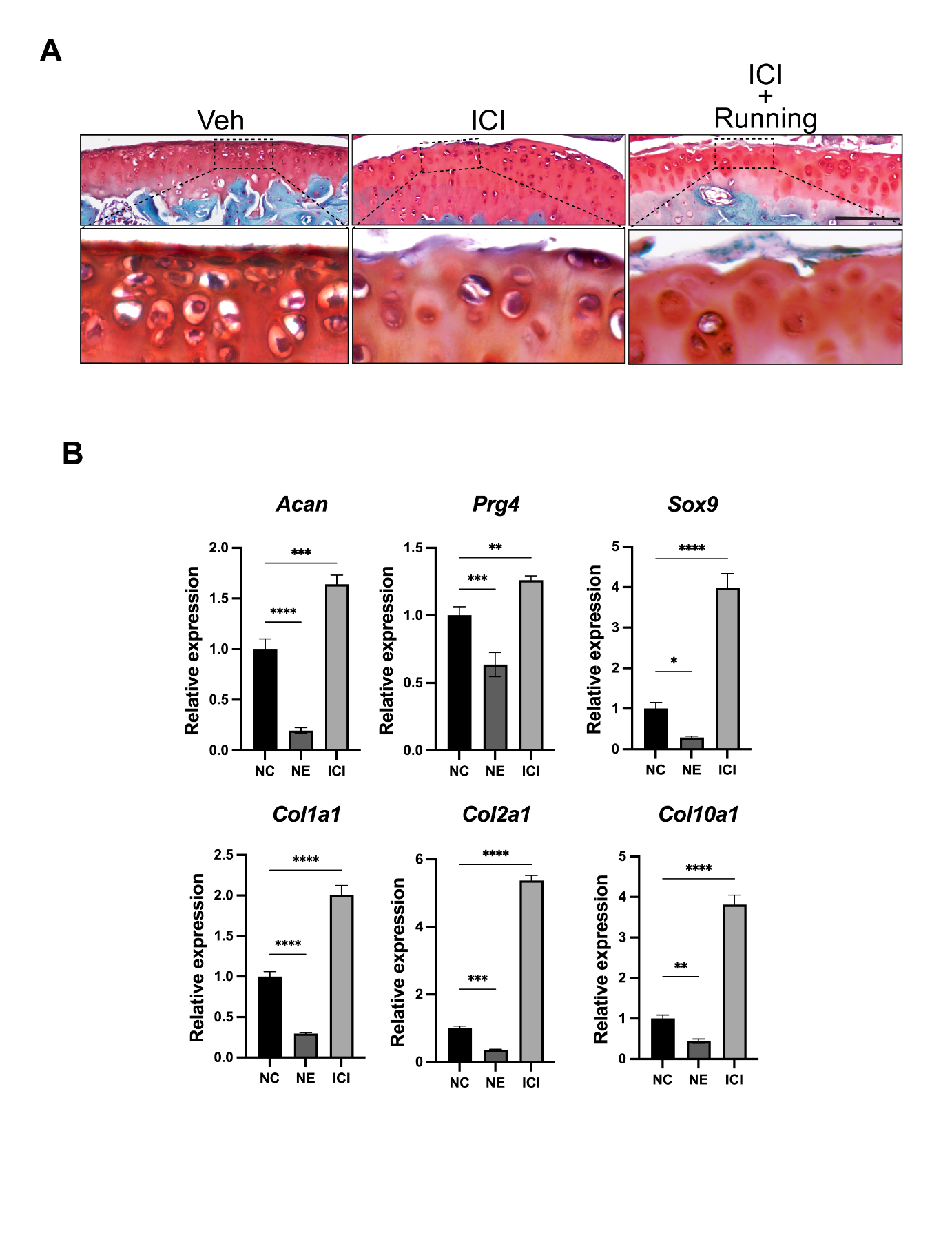


**Figure S7. NE inhibits SFZ chondrocyte activity and injection of Adrb2 antagonist ICI disrupts the SFZ membrane.** (**A**) Images of Safranin O/fast green staining of the joint after daily treatment with the Adrb2 specific antagonist, ICI-118551 (ICI) for 3 weeks then the mice were subjected to 7 days of voluntary treadmill running. (**B**) RT-qPCR analysis of relative expression of chondrogenic marker genes in superficial chondrocytes treated with NE and ICI for 3 hours. *n =* 3 per time point.


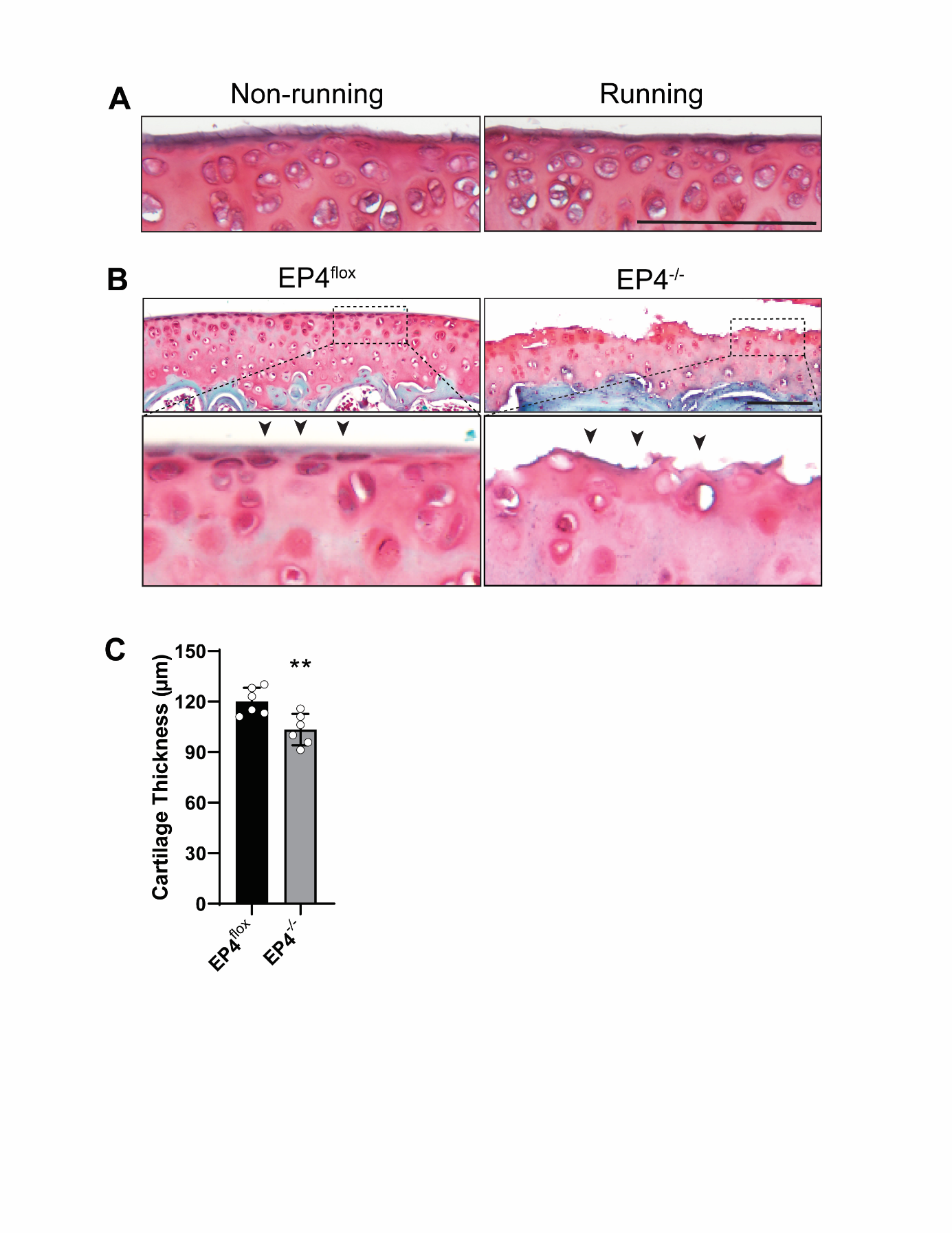


**Figure S8. Comparison of female mice in different experiments.** (**A**) 8-week-old female mice were running voluntarily on a treadmill for 7 consecutive days and their articular cartilages were analyzed using Safranin O/fast green staining. (**B, C**) Representative Safranin O/fast green staining of the articular cartilage of EP4^flox^ and EP4*^-/-^* mice (B) and their quantitation (V). *n =* 6 each group. Scale bar, 100 μm.

**Table S1.** **Antibody information**

| **Antibody** | **Catalog** | **Vendor** | **Use** | **Dilution** |
| --- | --- | --- | --- | --- |
| ADRB2 | ab182136 | Abcam | IHC, WB | 1:400 |
| Tyrosine Hydroxylase (TH) | AB125 | Millipore Sigma | IHC | 1:200 |
| Vasopressin (AVP) | 403004 | Synaptic System | IHC | 1:500 |
| pSmad2 | Ab280888  18338S | Abcam  Cell Signaling Technology | IHC, WB, ICC | 1:200, 1:1000, 1:500 |
| ADRB2 | NBP3-12006 | Novus Biologicals | ICC | 1:500 |
| TβRII | sc-17799 | Santa Cruz Biotechnology | IP, ICC, WB | 1:500, 1:1000 |
| Smad2 | 5339S | Cell Signaling Technology | WB | 1:1000 |
| CGRP | ab36001 | Abcam | IHC | 1:200 |
| PKA | 4782S | Cell Signaling Technology | WB | 1:1000 |
| Phospho-ERK1/ERK2 | MA5-33180 | Thermo Fisher Scientific | WB | 1:1000 |
| ERK1/2 | MA5-35579 | Thermo Fisher Scientific | WB | 1:1000 |
| β-Actin | 3700S | Cell Signaling Technology | WB | 1:3000 |
| Donkey Anti-Goat IgG H&L (Alexa Fluor® 647) | ab150135 | Abcam | IHC | 1:200 |
| Goat Anti-Rabbit IgG H&L (Alexa Fluor® 647) | ab150083 | Abcam | IHC | 1:200 |
| Goat Anti-Mouse IgG H&L (Alexa Fluor® 647) | ab150115 | Abcam | IHC | 1:200 |
| Donkey Anti-Goat IgG H&L (Alexa Fluor® 594) | 705-586-147 | The Jackson Laboratory | IHC | 1:200 |
| Donkey Anti-Rabbit IgG H&L (Alexa Fluor® 594) | 711-586-152 | The Jackson Laboratory | IHC | 1:200 |
| Donkey Anti-Mouse IgG H&L (Alexa Fluor® 594) | 715-585-151 | The Jackson Laboratory | IHC | 1:200 |
| Donkey Anti-Goat IgG H&L (Alexa Fluor® 488) | 705-546-147 | The Jackson Laboratory | IHC | 1:200 |
| Donkey Anti-Rabbit IgG H&L (Alexa Fluor® 488) | 711-546-152 | The Jackson Laboratory | IHC | 1:200 |
| Donkey Anti-Mouse IgG H&L (Alexa Fluor® 488) | 715-546-151 | The Jackson Laboratory | IHC | 1:200 |
| Swine Anti-Rabbit Immunoglobulins/HRP | P0217 | Dako | WB | 1:3000 |
| Rabbit Anti-Mouse Immunoglobulins/HRP | P0260 | Dako | WB | 1:3000 |

IHC, immunohistochemistry; ICC, Immunocytochemistry; WB, western blot

**Table S2: Chemicals information**

| **Name** | **Catalog** | **Vendor** |
| --- | --- | --- |
| SW033291 | S7900 | Selleck Chemicals |
| ICI 118,551 hydrochloride | 0821 | Tocris Bioscience |
| Recombinant Human TGF-beta 1 Protein | 7754-BH-100/CF | R & D Systems |
| L-Norepinephrine hydrochloride | 74480 | Sigma-Aldrich |
| NHS-FITC | 46410 | Thermo Fisher |

**Table S3: PCR primers**

| **Name** | **Forward** | **Reverse** |
| --- | --- | --- |
| *Prg4* | 5′-TGGTAAGCCAGTGGATGGACTG-3′ | 5′-GGTAATTCTGCGTGGTGGAGATG-3′ |
| *β-Actin* | 5′-CATTGCTGACAGGATGCAGAAGG-3′ | 5′-TGCTGGAAGGTGGACAGTGAGG-3′ |
| *Col1a1* | 5′- CCTCAGGGTATTGCTGGACAAC-3′ | 5′-CAGAAGGACCTTGTTTGCCAGG-3′ |
| *Col10a1* | 5′- GTACCAAACGCCCACAGGCATA-3′ | 5′-GGACCAGGAATGCCTTGTTCTC-3′ |
| *Acan* | 5'-CAGGCTATGAGCAGTGTGATGC-3' | 5'-GCTGCTGTCTTTGTCACCCACA-3' |
| *Col2a1* | 5'-GCTGGTGAAGAAGGCAAACGAG-3' | 5'-CCATCTTGACCTGGGAATCCAC-3' |
| *Sox9* | 5'-CACACGTCAAGCGACCCATGAA-3' | 5'-TCTTCTCGCTCTCGTTCAGCAG-3' |

**Table S4:** **Commercial Kit**

| **Kit name** | **Catalog** | **Vendor** |
| --- | --- | --- |
| PGE2 ELISA kit | 514010 | Cayman |
| NE ELISA kit | KA3836 | Novus Biologicals |
| Pierce™ Classic IP kit | 26146 | Thermo Fisher |
| PrimeScript™ RT Master Mix kit | RR036A | Takara |

**Table S5: Software**

| **Name** | **Version** | **RRID** |
| --- | --- | --- |
| ImageJ | v1.54g | SCR_003070 |
| Imaris | v10.0 | SCR_007370 |
| Graph Pad | v8.0 | SCR_002798 |
| Zen blue | v3.2 | SCR_013672 |
