# Supplementary material for: Hypothalamus Regulates Anabolic Metabolism of Articular Cartilage Superficial Chondrocytes through PGE2 Skeletal Interoception
Source: Adv Sci (Weinh). 2025 Mar 26;12(19):2501039. doi: 10.1002/advs.202501039 (PMC12097074; doi:10.1002/advs.202501039)

ADVANCED SCIENCE
Open Access

Supporting Information

for *Adv. Sci.*, DOI 10.1002/advs.202501039

Hypothalamus Regulates Anabolic Metabolism of Articular Cartilage Superficial Chondrocytes through PGE2 Skeletal Interoception

*Ziyi Wang, Xuequan Han, Jiawen Xu, Weixin Zhang, Kalp Patel, Jinjian Zheng, Mei Wan, Junying Zheng and Xu Cao**

Figure 1 TH staining

Non-running Group

Animal 1-1
(main figure)

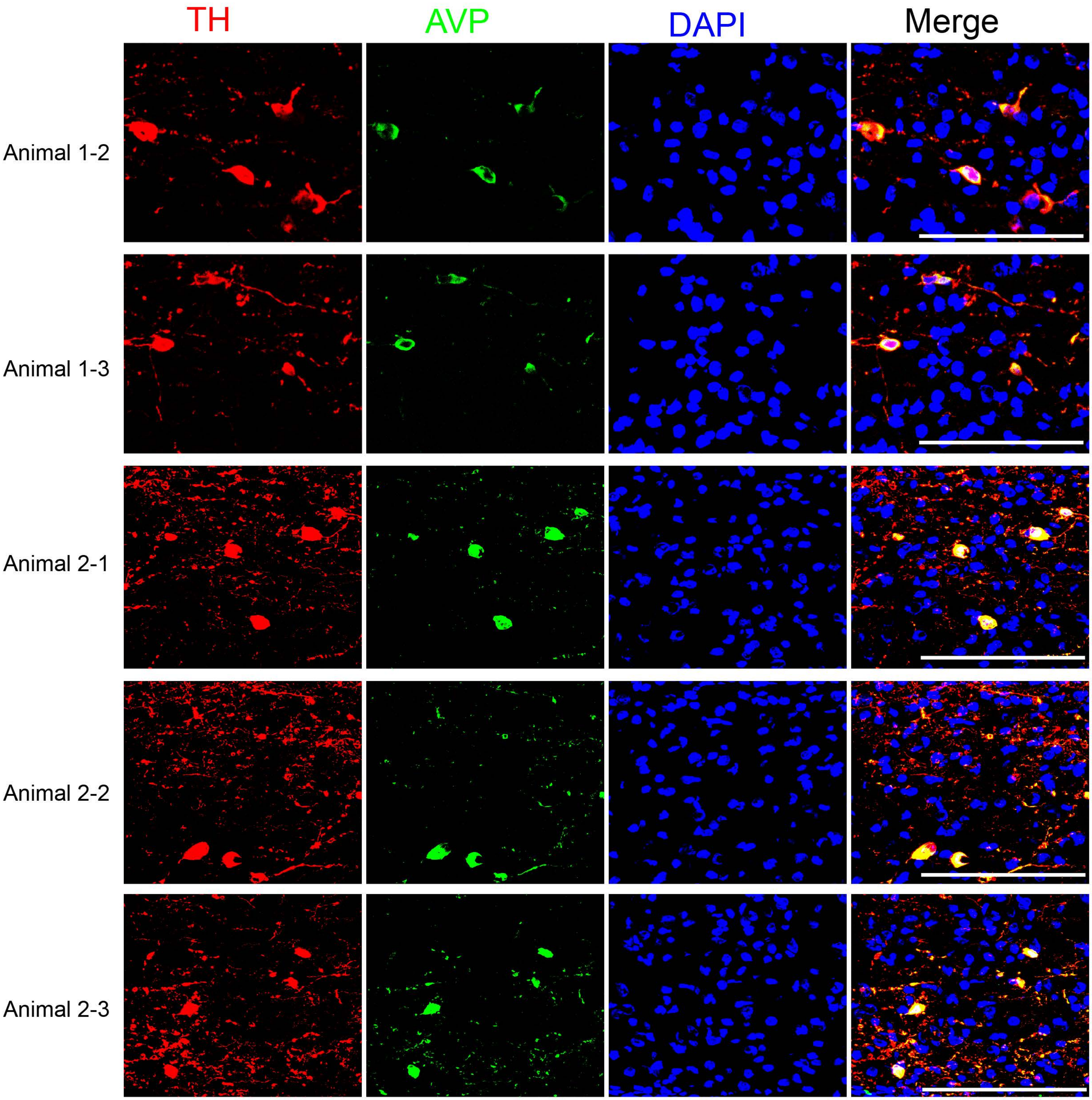

Figure 1 TH staining

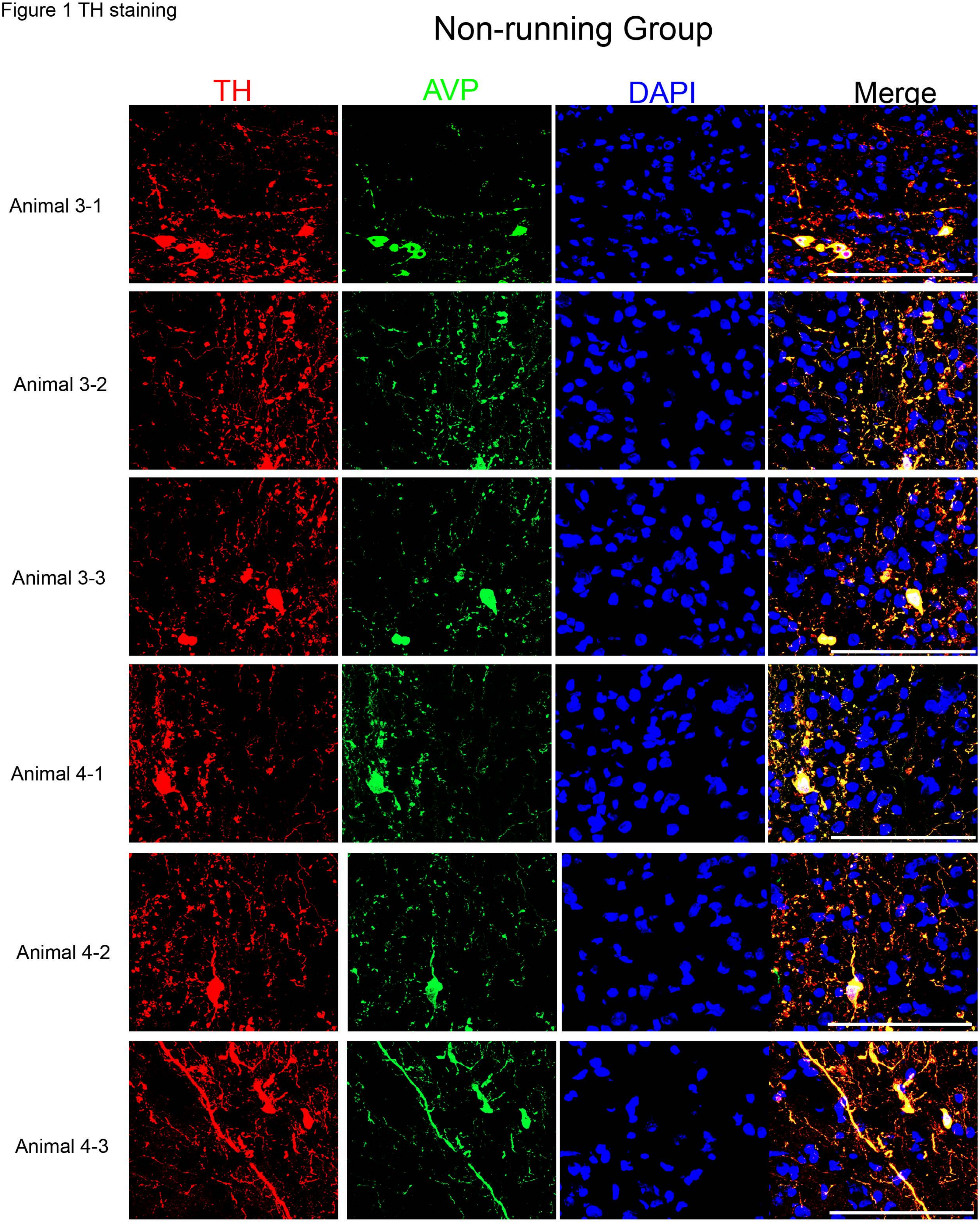

Figure 1 TH staining

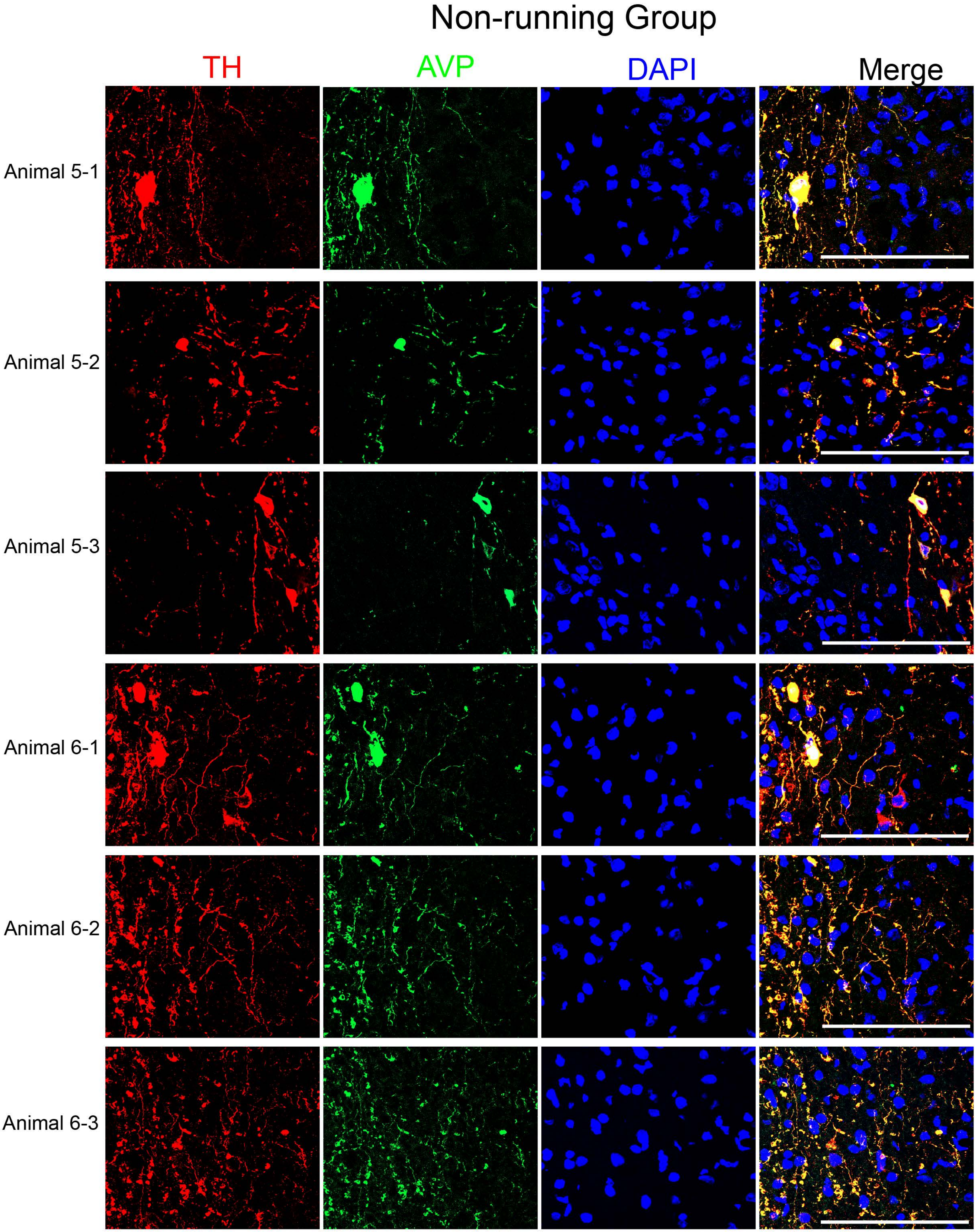

Figure 1 TH staining

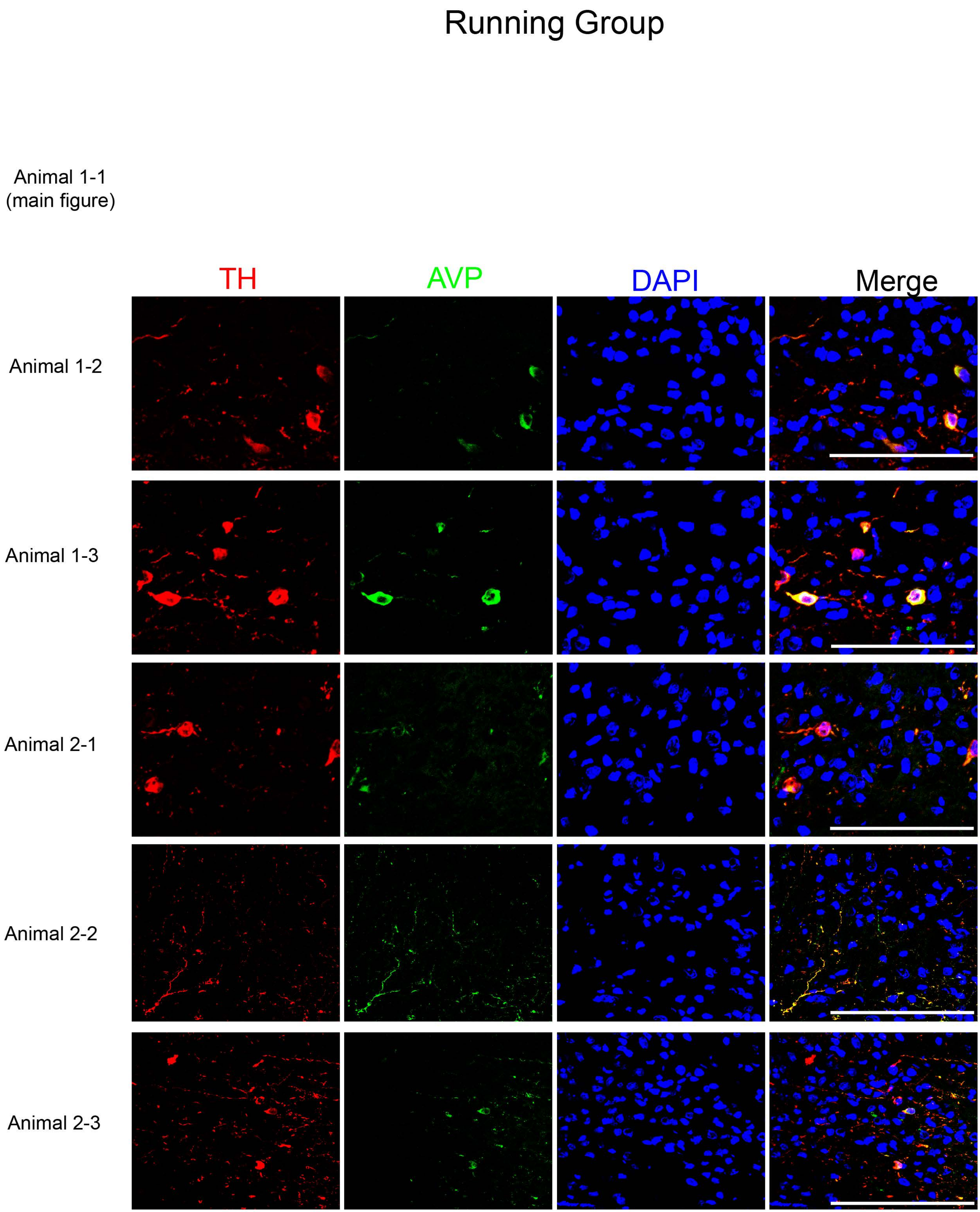

Figure 1 TH staining

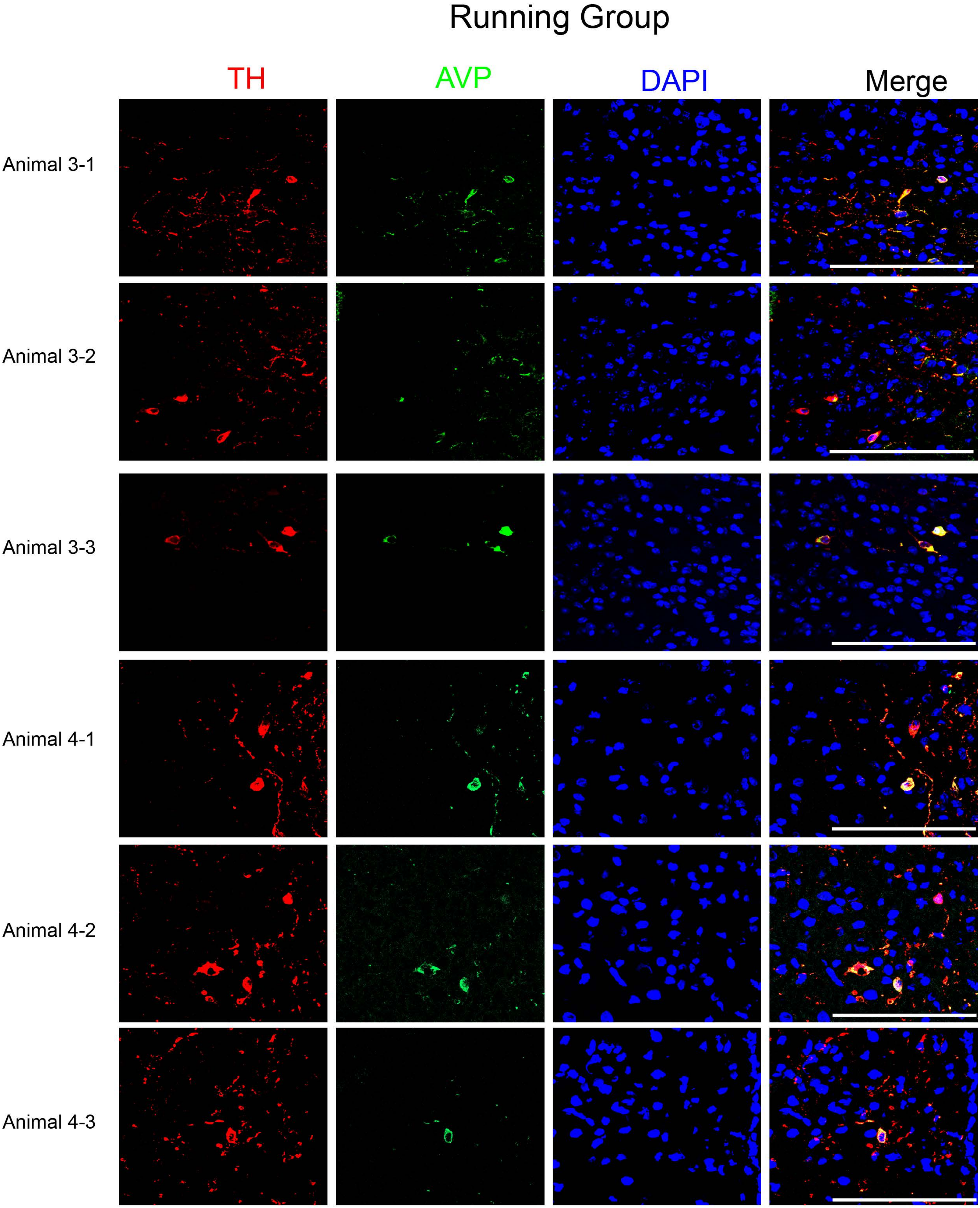

Figure 1 TH staining

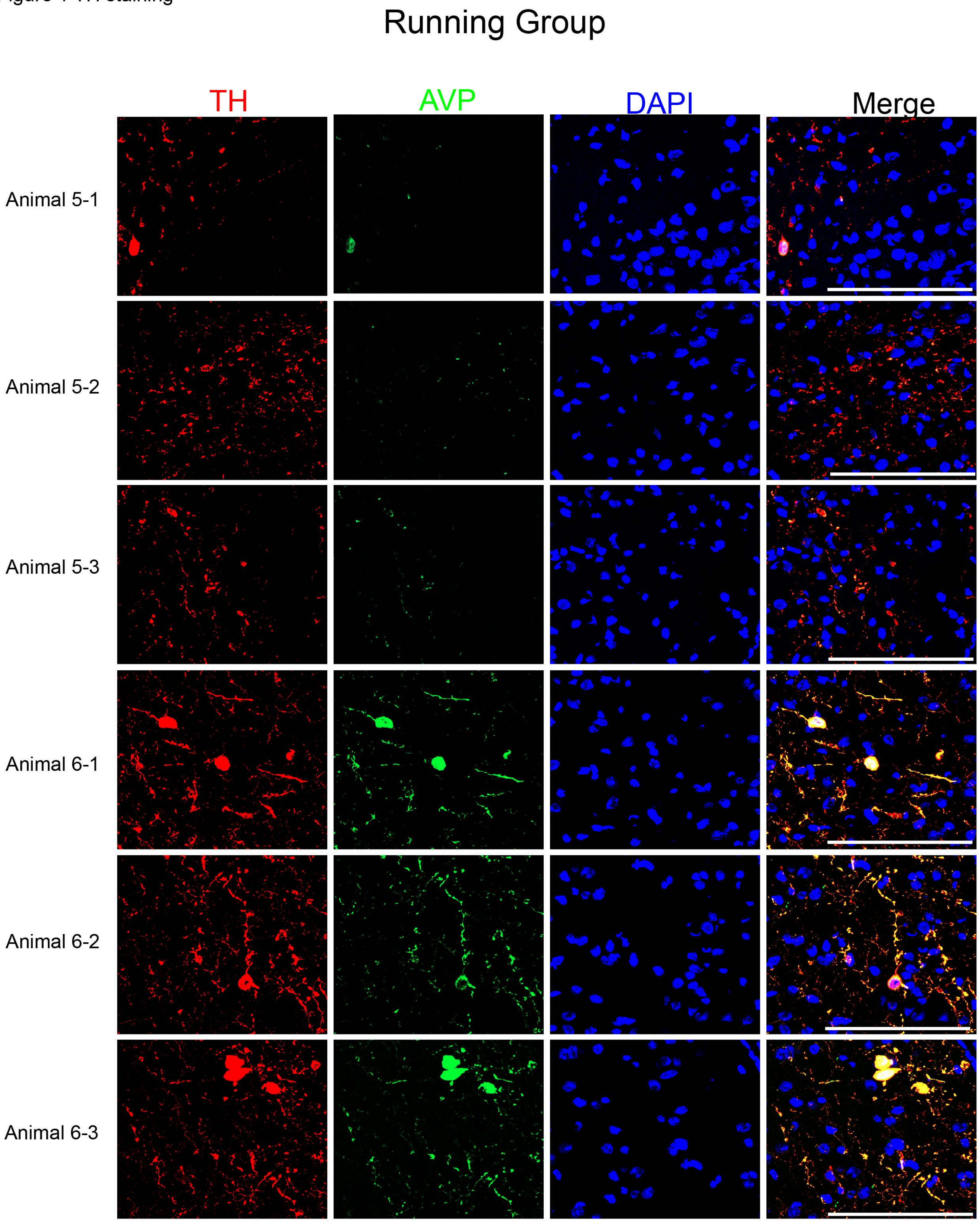

Figure 2 TH staining

EP4^{flox}Group

Animal 1-1
(main figure)

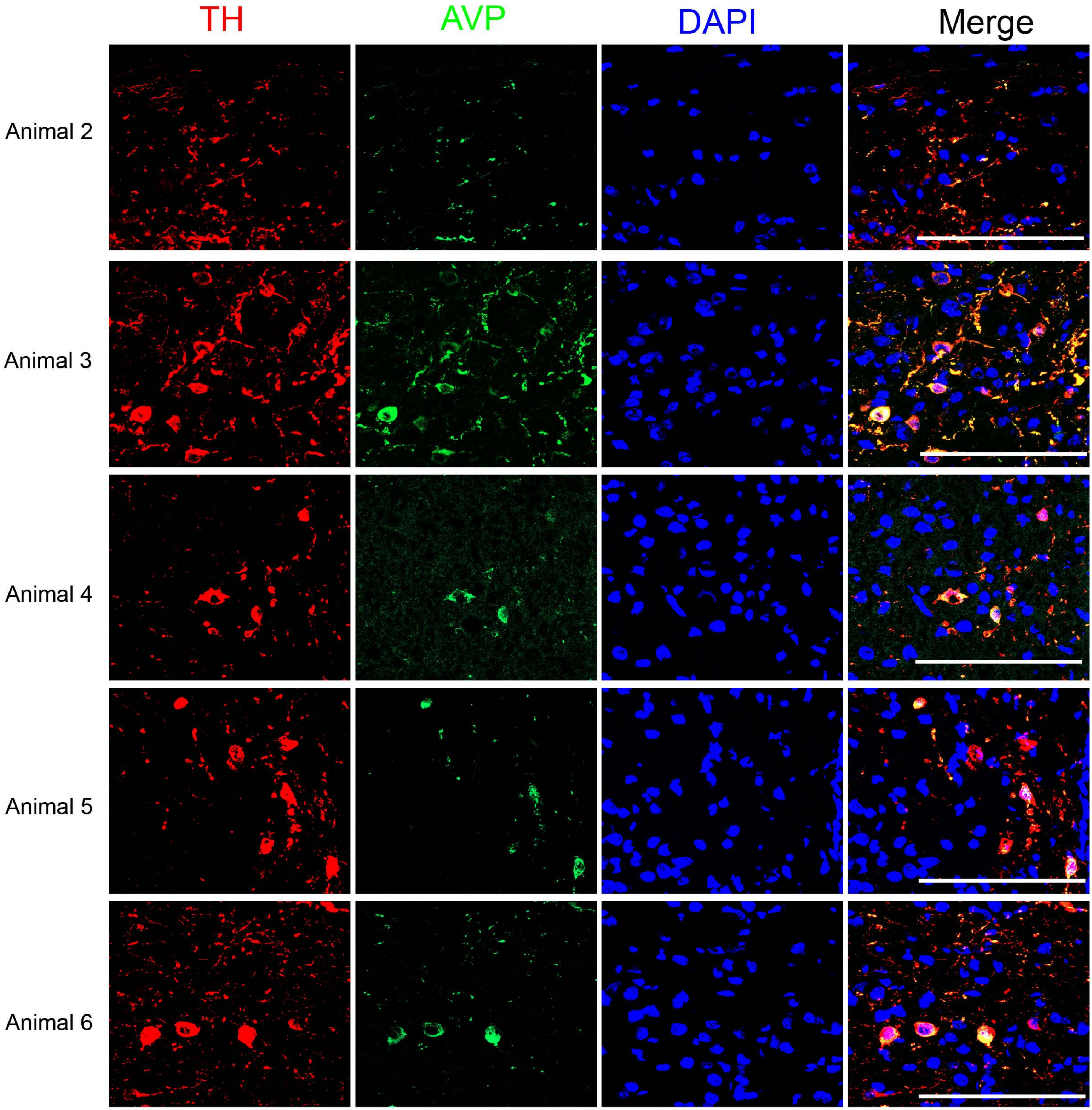

Figure 2 TH staining

EP4$^{-/-}$Group

Animal 1-1
(main figure)

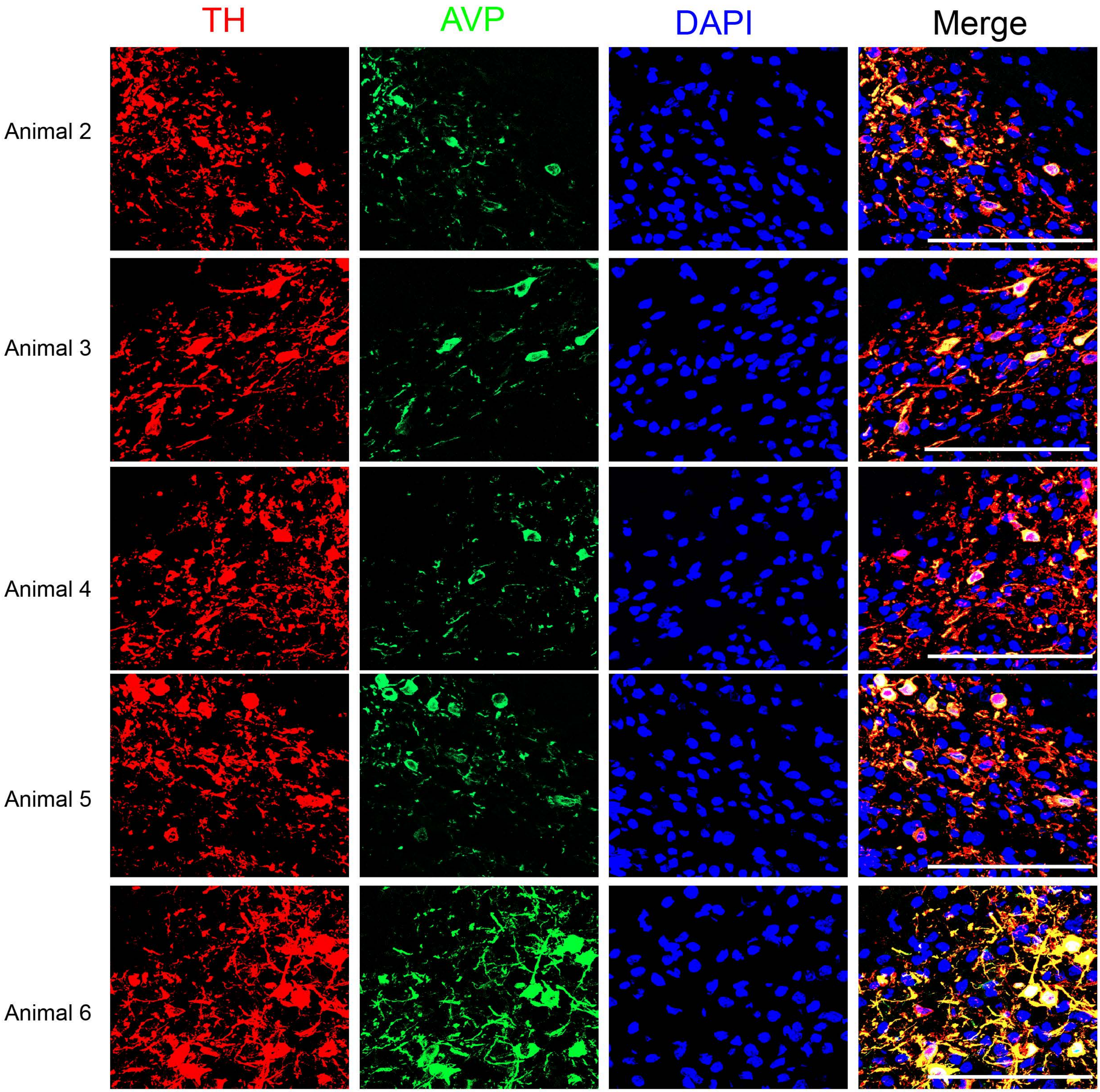

Figure 3 TH staining

EP4^{flox} Veh Group

Animal 1-1
(main figure)

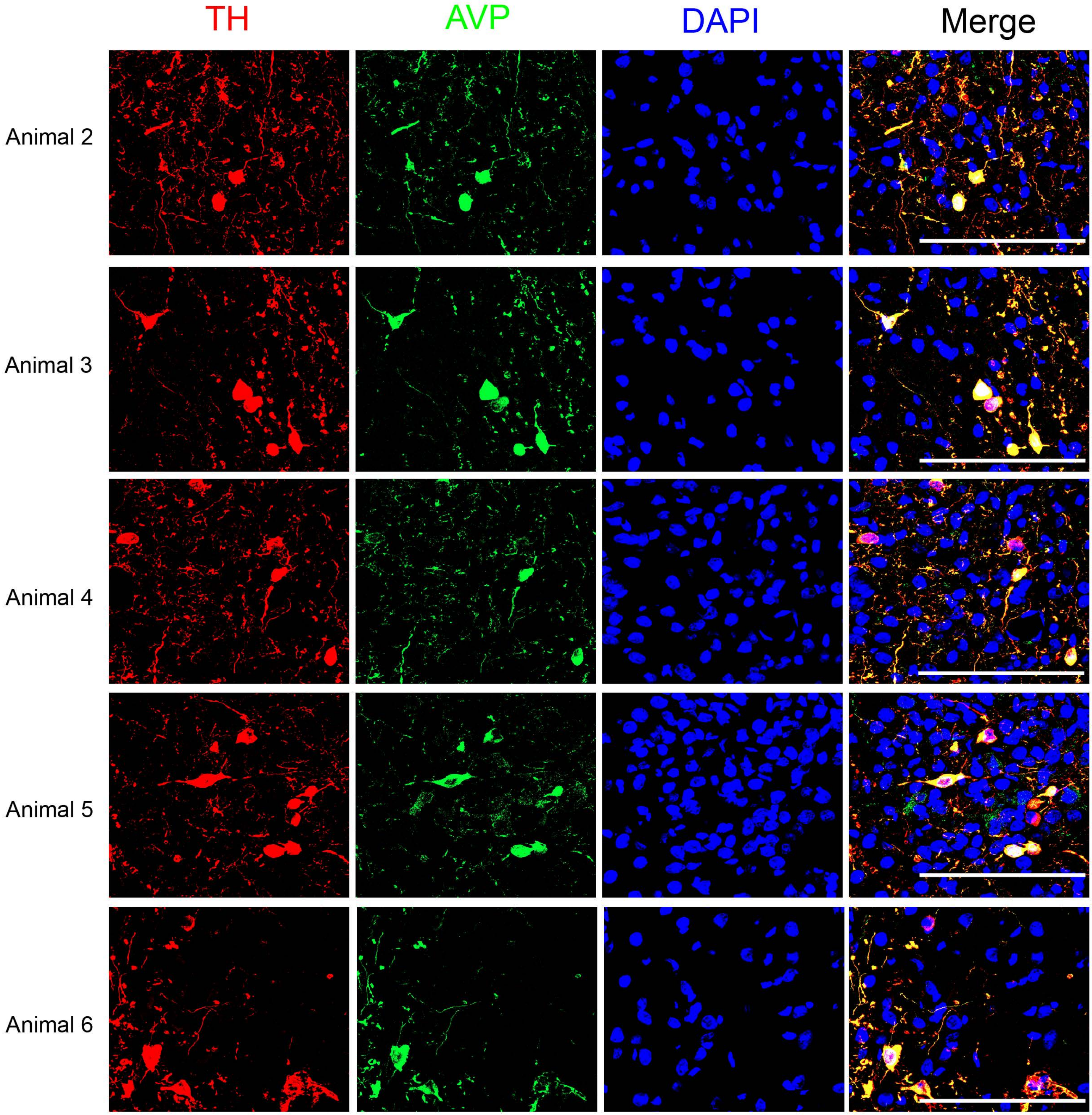

Figure 3 TH staining

EP4^{flox} SW Group

Animal 1-1
(main figure)

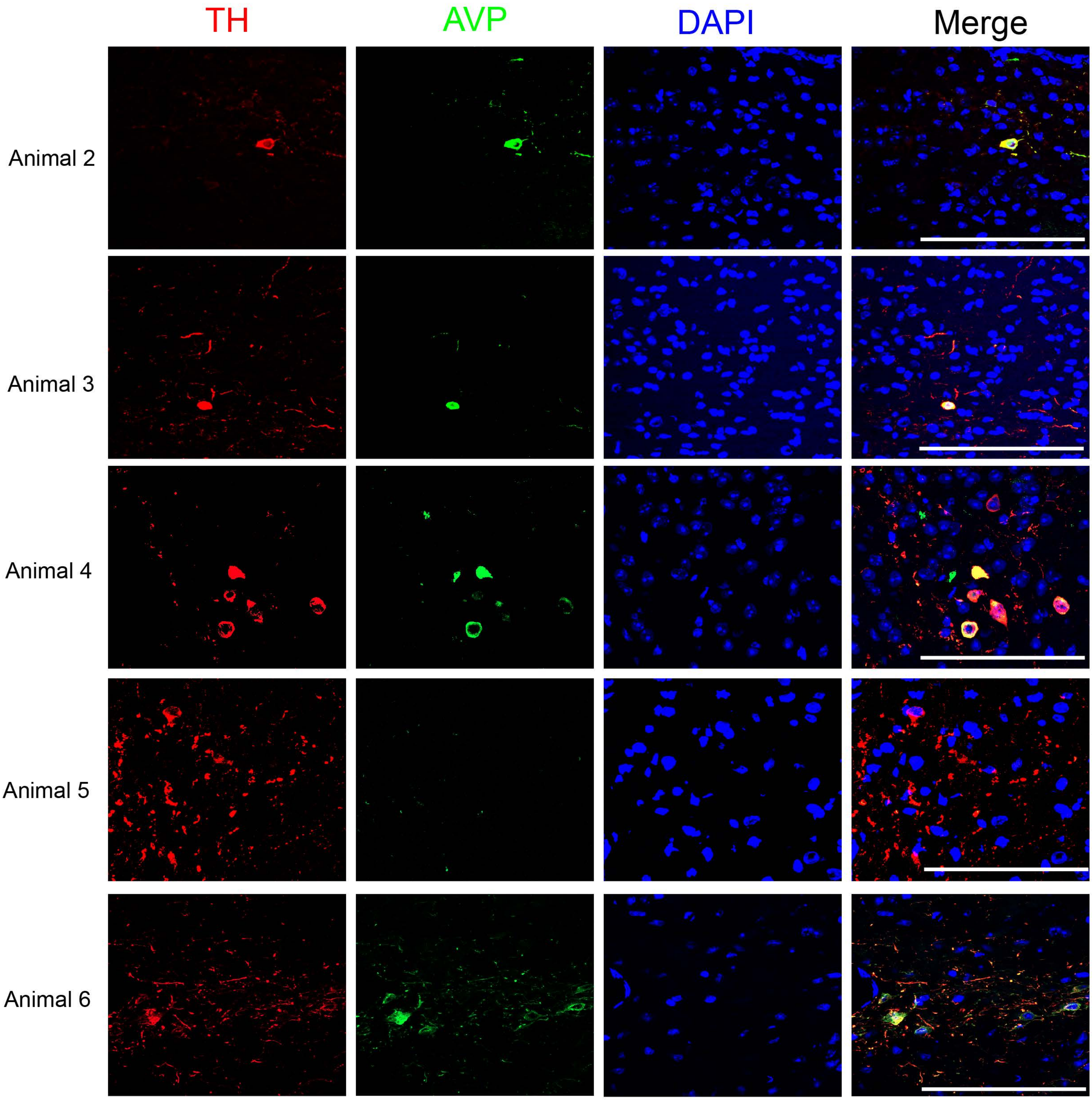

Figure 3 TH staining

EP4$^{-/-}$ Veh Group

Animal 1-1
(main figure)

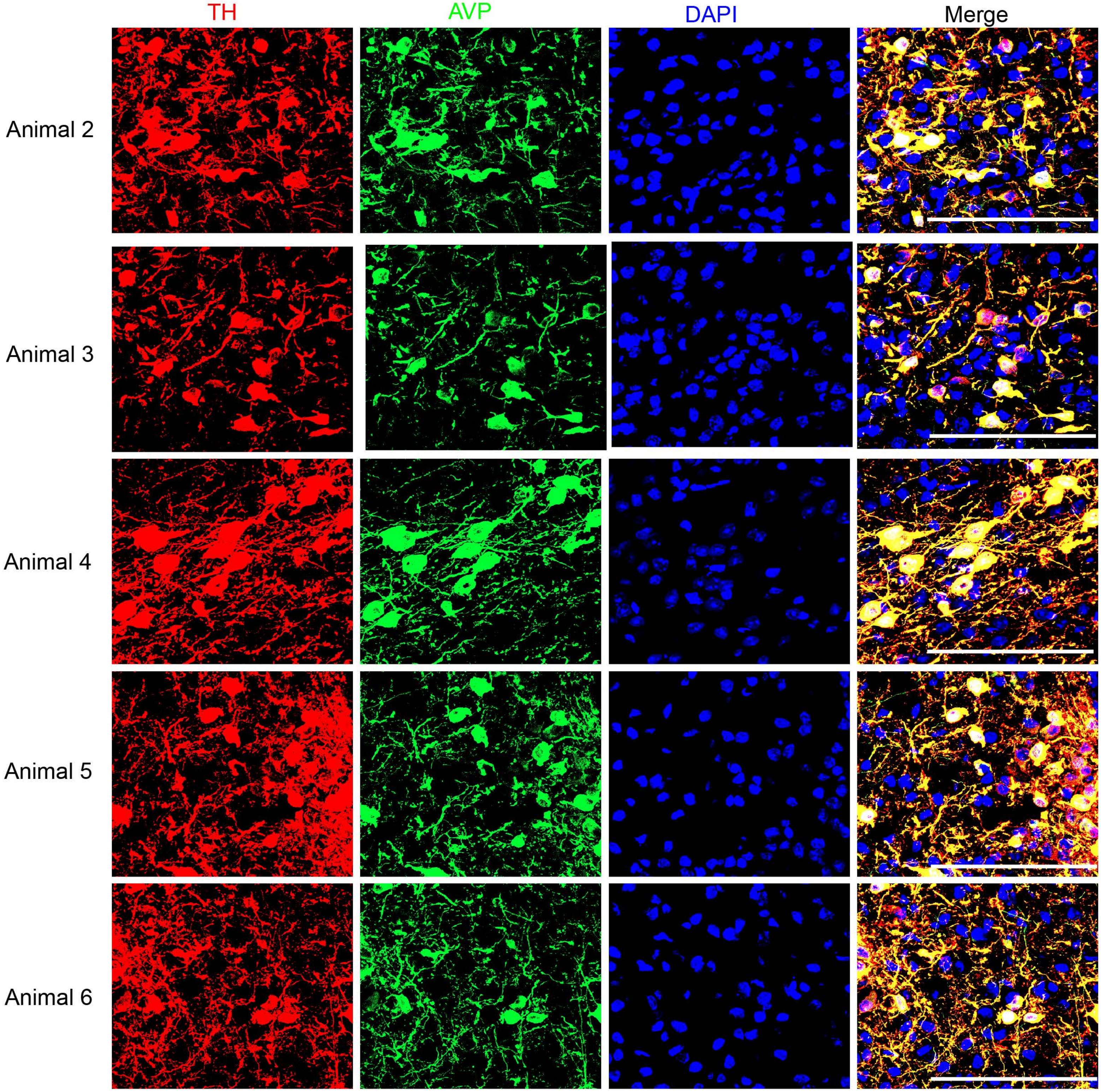

Figure 3 TH staining

EP4$^{-/-}$ SWGroup

Animal 1-1
(main figure)

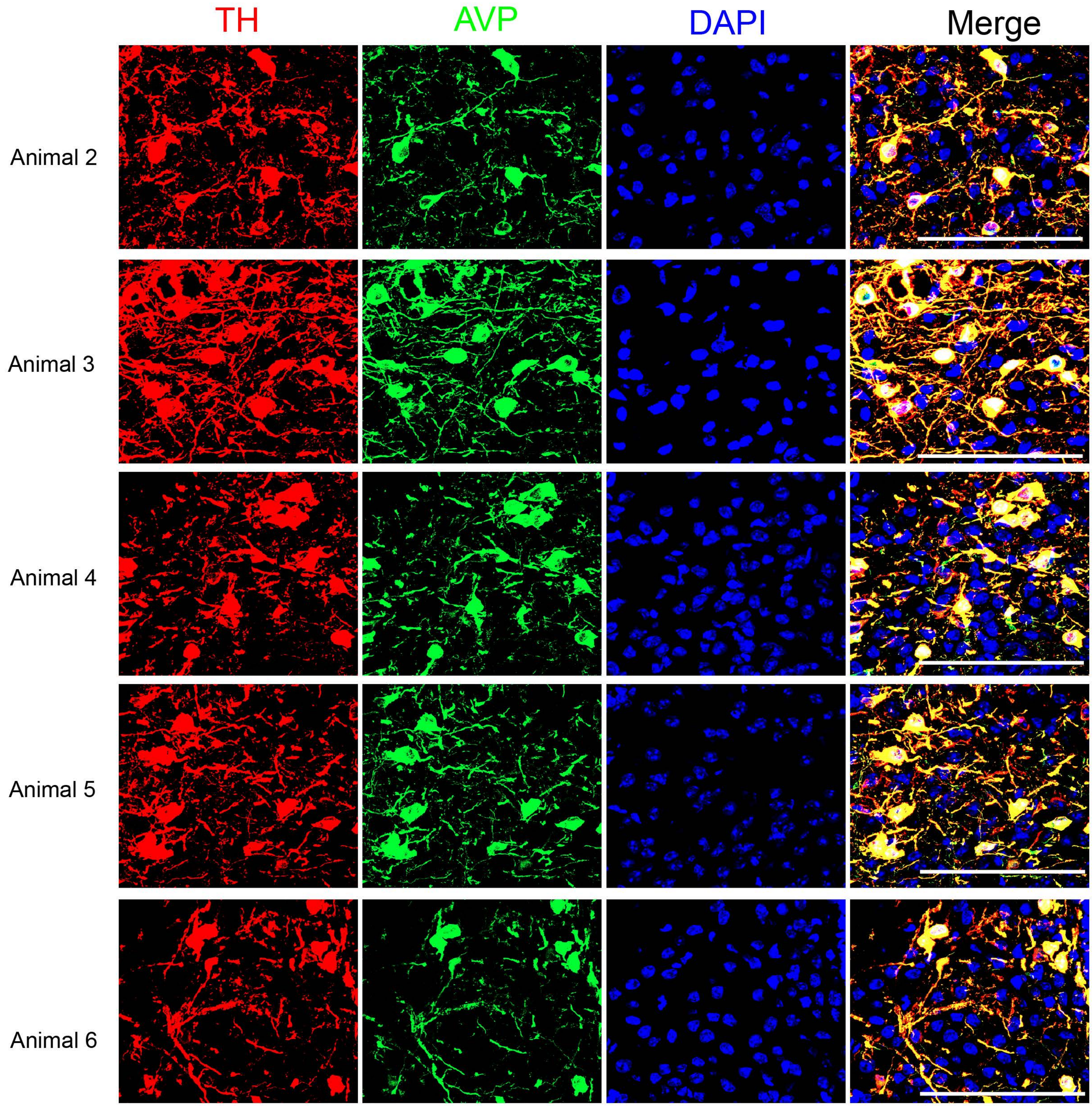

Figure 4 TH staining

AAV-Ctrl Group

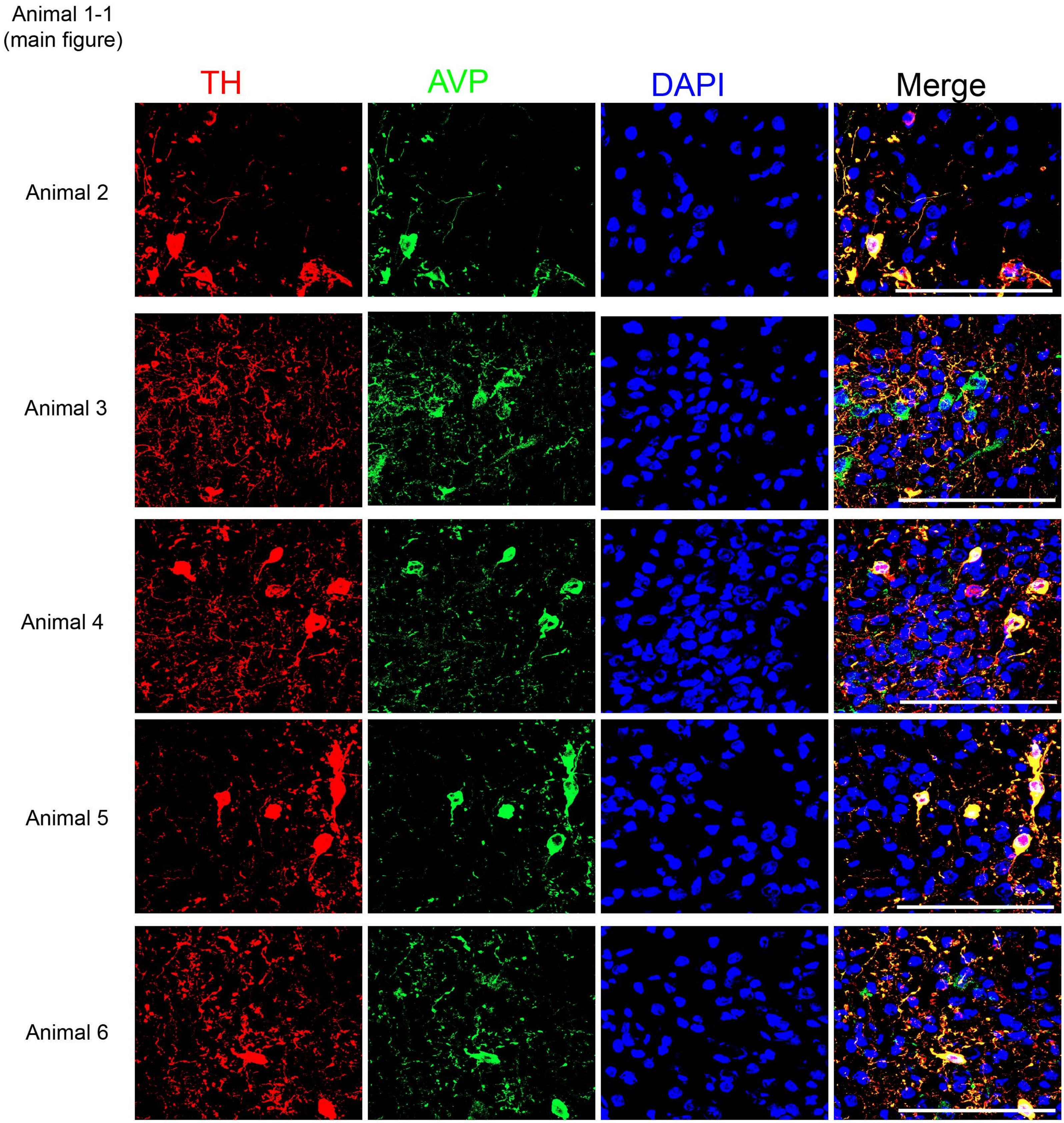

Figure 4 TH staining

AAV-shTH Group

Animal 1-1
(main figure)

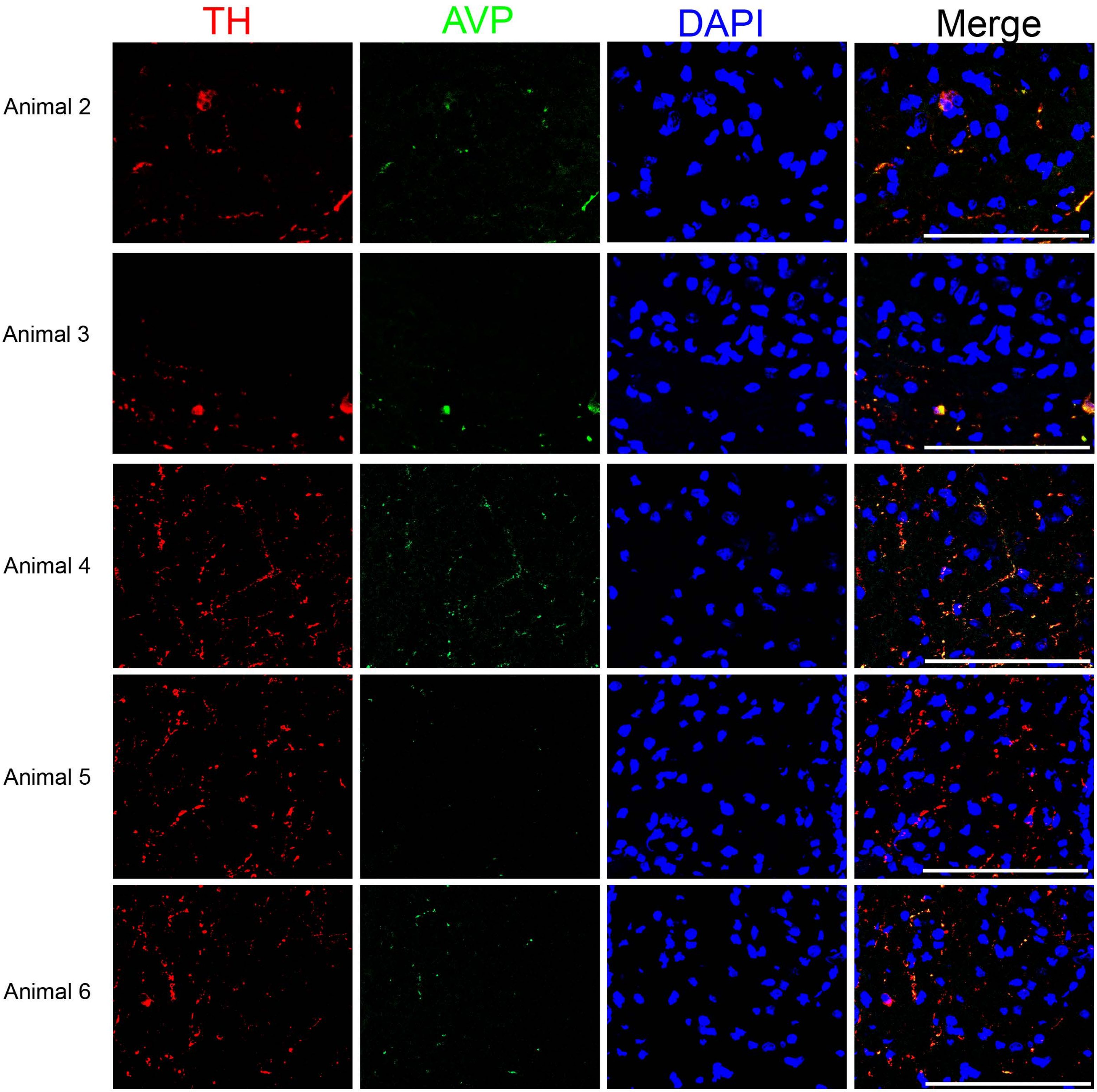

Figure 5 TH staining

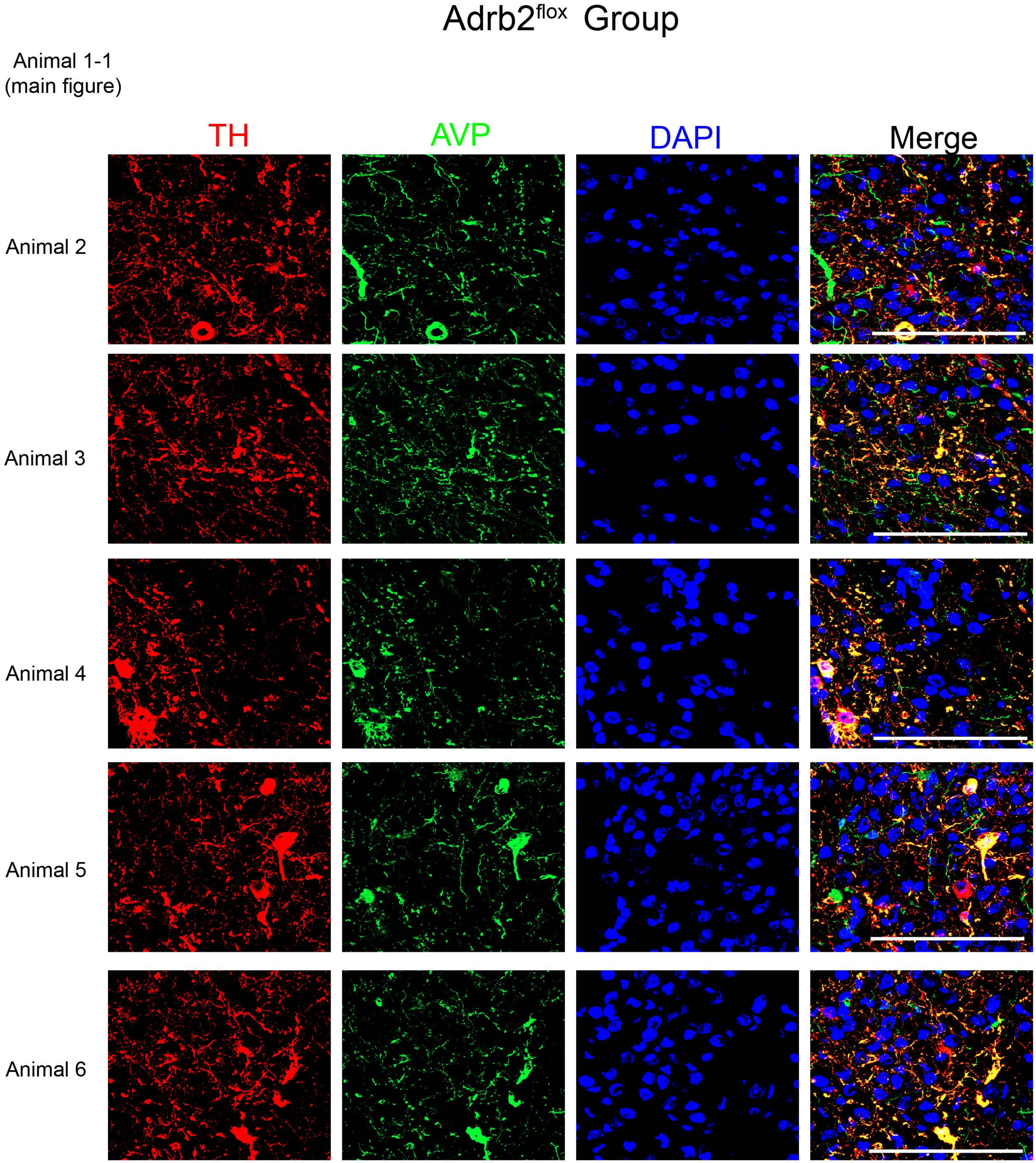

Figure 5 TH staining

Adrb2$^{-/-}$ Group

Animal 1-1
(main figure)

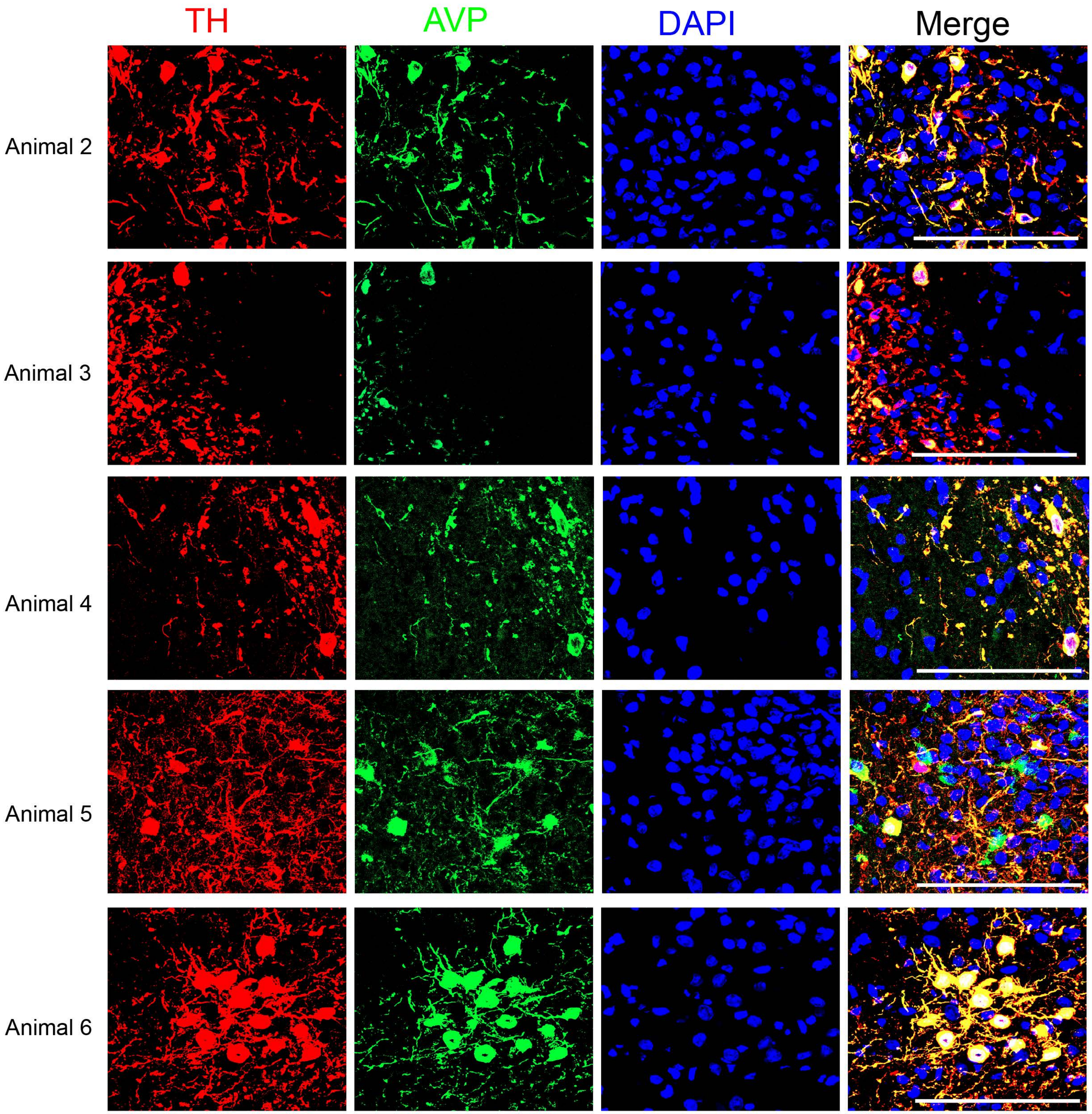

Supplement: Supplementary file 2 — Supporting Information [file ADVS-12-2501039-s003.pdf]
